# Functional connectivity in Parkinson’s disease candidates for deep brain stimulation

**DOI:** 10.1038/s41531-021-00268-6

**Published:** 2022-01-10

**Authors:** Luigi Albano, Federica Agosta, Silvia Basaia, Camilla Cividini, Tanja Stojkovic, Elisabetta Sarasso, Iva Stankovic, Aleksandra Tomic, Vladana Markovic, Elka Stefanova, Pietro Mortini, Vladimir S. Kostic, Massimo Filippi

**Affiliations:** 1 Neuroimaging Research Unit, Division of Neuroscience, IRCCS Ospedale San Raffaele, Milan, Italy; 2 Neurosurgery and Gamma Knife Radiosurgery Unit, IRCCS Ospedale San Raffaele, Milan, Italy; 3 Vita-Salute San Raffaele University, Milan, Italy; 4 Neurology Unit, IRCCS Ospedale San Raffaele, Milan, Italy; 5 Clinic of Neurology, Faculty of Medicine, University of Belgrade, Belgrade, Serbia; 6 Neurorehabilitation Unit, IRCCS Ospedale San Raffaele, Milan, Italy; 7 Neurophysiology Service, IRCCS Ospedale San Raffaele, Milan, Italy

**Keywords:** Parkinson's disease, Parkinson's disease

## Abstract

This study aimed to identify functional neuroimaging patterns anticipating the clinical indication for deep brain stimulation (DBS) in patients with Parkinson’s disease (PD). A cohort of prospectively recruited patients with PD underwent neurological evaluations and resting-state functional MRI (RS-fMRI) at baseline and annually for 4 years. Patients were divided into two groups: 19 patients eligible for DBS over the follow-up and 41 patients who did not meet the criteria to undergo DBS. Patients selected as candidates for DBS did not undergo surgery at this stage. Sixty age- and sex-matched healthy controls performed baseline evaluations. Graph analysis and connectomics assessed global and local topological network properties and regional functional connectivity at baseline and at each time point. At baseline, network analysis showed a higher mean nodal strength, local efficiency, and clustering coefficient of the occipital areas in candidates for DBS over time relative to controls and patients not eligible for DBS. The occipital hyperconnectivity pattern was confirmed by regional analysis. At baseline, a decreased functional connectivity between basal ganglia and sensorimotor/frontal networks was found in candidates for DBS compared to patients not eligible for surgery. In the longitudinal analysis, patient candidate for DBS showed a progressively decreased topological brain organization and functional connectivity, mainly in the posterior brain networks, and a progressively increased connectivity of basal ganglia network compared to non-candidates for DBS. RS-fMRI may support the clinical indication to DBS and could be useful in predicting which patients would be eligible for DBS in the earlier stages of PD.

## Introduction

Parkinson’s disease (PD) is one of the most common neurodegenerative disorders affecting millions of people worldwide. It is characterized by motor symptoms, such as resting tremor, rigidity, bradykinesia, as well as non-motor manifestations including autonomic dysfunction, behavioral and cognitive impairments^1^. The progressive death of the pigmented neurons of substantia nigra pars compacta is the principal pathological hallmark^2^. Several treatments, including medications, surgery and supportive therapies, are currently available to help relieve symptoms and maintain an acceptable quality of life^1^.

Deep brain stimulation (DBS) has become a well-established therapy for PD and few treatments are as effective as DBS for controlling the troubling motor symptoms of PD, levodopa-induced dyskinesia, and quality of life^3–5^. Currently, indications of DBS for PD are based on general and clinical characteristics. The following factors have to be carefully assessed before recommending surgery to a given patient: disease duration (of at least 4 years according to the Earlystim clinical trial)^3,5^, levodopa responsiveness, type and severity of motor symptoms, cognitive and psychiatric issues, comorbidities, and brain magnetic resonance imaging (MRI) findings^6,7^. Even with established indications to DBS, key questions remain unanswered. It is not yet clear, in fact, how the clinical benefit with DBS is achieved, why some symptoms (e.g., tremor) respond more quickly than others, and why the therapeutic benefits of DBS still vary among PD patients. The correct selection of the optimal candidate to DBS for PD may play a significant role in this variability.

The recent advances in the field of neuroimaging are continuously expanding our knowledge of the brain areas and circuits underlying the clinical expression of PD^8–11^. Resting-state (RS) functional magnetic resonance imaging (fMRI), in particular, can provide a detailed description of how the disease alters the functional brain organization and further allow to make new hypotheses about PD physiopathology^11,12^. More recently, RS-fMRI studies demonstrated that functional connectivity patterns may identify different clinical clusters of PD or predict motor benefit from DBS, and that altered functional brain networks correlate with PD motor and cognitive severity^9,13–17^.

To our knowledge, there are no studies investigating functional neuroimaging features in PD patients eligible for DBS before becoming clinical candidates for surgical treatment, probably due to the lack of large cohorts of PD patients prospectively followed with brain MRI. For this purpose, in this study, we investigated functional connectivity patterns in two cohorts of PD patients over time, including both patients eligible for DBS over time and cases with comparable disease duration and stage but not meeting the criteria to undergo DBS treatment; patients selected as candidates for DBS did not undergo surgery at this stage. We aimed to investigate brain network differences before patients became eligible for DBS, how they change over time in both groups, and to look into identifying an early predictive biomarker of indication to DBS.

## Results

### Demographic and clinical features

**Fig. 1:**
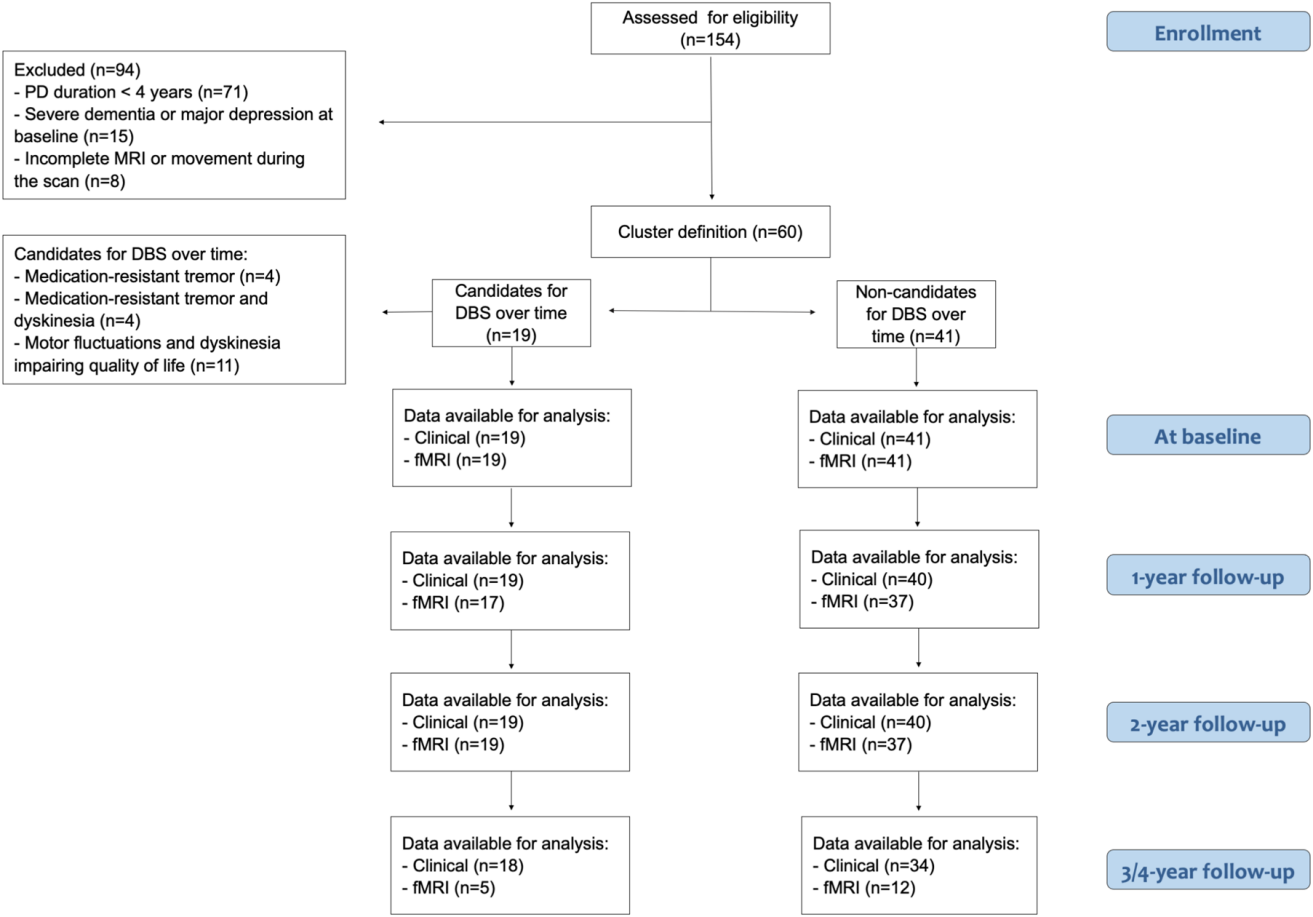
Flowchart illustrating the inclusion/exclusion of participants in the study. Chart data for 154 patients with idiopathic Parkinson’s disease (PD) prospectively recruited at the Clinic of Neurology, Faculty of Medicine, University of Belgrade, Belgrade, Serbia. According to inclusion and exclusion criteria, 60 patients were included in the study. The study cohort was then divided in two groups: 19 patients eligible for DBS treatment and 41 patients who did not meet the criteria to undergo DBS over time. Patients were assessed by clinical, cognitive/behavioral, and brain MRI evaluations at study entry and every year for a maximum of 4 years. DBS deep brain stimulation, fMRI functional MRI, PD Parkinson’s disease.

Nineteen PD patients became eligible for DBS over the follow-up, whereas 41 patients did not meet the criteria to undergo future surgery. Candidates for DBS over the follow-up became eligible on average 26.53 ± 8.45 months from the study entry (range, 12–48), according to the following clinical indications: medication-resistant tremor in 4/19 patients (21.05%), motor fluctuations, and dyskinesia impairing quality of life in 11/19 patients (57.9%), and medication-resistant tremor and dyskinesia in the remaining 4/19 patients (21.05%) (Fig. 1).

Demographic and clinical characteristics of the study sample at baseline and their changes over time are described in Table 1. No differences in age and sex were found among the groups. Both patient groups had lower education and worse cognitive and behavioral performances relative to controls (Table 1 and Supplementary Table 1). Patients eligible for DBS and those not eligible did not differ in terms of education, age at onset, PD duration, site of onset, and Hoehn and Yahr (HY) score^18^ at baseline. At baseline, candidates for DBS were characterized by more severe motor signs and symptoms than non-candidates (Table 1). More specifically, the Unified PD Rating Scale (UPDRS)^19^ total, part II, part III and part IV scores were worse in the former than in the latter group of patients (Table 1). Over follow-up, both PD groups showed motor clinical progression (i.e., UPDRS total, part II, part III, part IV) and increased levodopa equivalent daily dosage (LEDD)^20^ (Table 1). Furthermore, patients eligible for DBS showed a more severe worsening of UPDRS IV score relative to non-candidates for surgery. However, they did not undergo surgery at this stage. Cognitive and behavioral features were similar between the two PD groups at baseline, except for language which was more impaired in candidates relative to non-candidates for DBS over the follow-up (Supplementary Table 1). Over follow-up, both PD groups showed a progressive worsening of cognitive functions (mainly in the executive, attentive and visuospatial domains); only patients eligible for DBS were characterized by alterations of memory tests over time and they showed a more severe worsening of attentive functions relative to the other group (Supplementary Table 1).

**Table 1:** Demographic and clinical features of Parkinson’s disease patients and healthy controls.

Variables	HC	Candidates for DBS	Non-candidates	*p*: Candidates for DBS vs HC	*p*: Non-candidates vs HC	*p*: Candidates for DBS vs Non-candidates	*p*: for linear trend, Candidates for DBS	*p*: for linear trend, Non-candidates	*p*: for differential trend between PD groups
*N*	60	19	41	–	–	–	–	–	–
Age at MRI [years]	61.79 ± 8.98 (46.14–77.72)	61.09 ± 7.32 (48.86–73.59)	61.96 ± 6.38 (49.09–82.95)	1.00	1.00	1.00	–	–	–
Sex [Men/women]	29 (48.33)/31 (51.67)	11 (57.9)/8 (42.1)	25 (61)/16 (39)	0.32	0.15	0.52	–	–	–
Education [years]	13.52 ± 2.57 (8.00–16.00)	11.21 ± 3.06 (4–16)	12.34 ± 2.31 (8–17)	0.003	0.01	0.35	–	–	–
Handedness [right/left/both]	46 (92) 4 (8) 0 (0)	18 (94.7) 1 (5.3) 0 (0)	36 (87.8) 4 (9.8) 1 (2.4)	0.58	0.50	0.64	–	–	–
Age at onset [years]	–	52.05 ± 7.94 (42.0–64.0)	54.63 ± 6.45 (43.0–71.0)	–	–	0.19	–	–	–
Disease duration [years]	–	8.94 ± 5.55 (4.28–23.93)	7.42 ± 3.65 (4.00–16.86)	–	–	0.24	–	–	–
Family history [No/yes]	–	16 (84.2) 3 (15.8)	34 (82.9) 7 (17.1)	–	–	0.61	–	–	–
Side of onset [Right/left/both]	–	12 (63.2) / 7 (36.8) / 0 (0)	25 (61.0) / 15 (36.6) / 1 (2.4)	–	–	0.79	–	–	–
Hoehn & Yahr	–	2.32 ± 0.74 (1–3)	1.94 ± 0.60 (1–3)	–	–	0.19	<0.001	<0.001	0.85
UPDRS total	–	65.00 ± 17.59 (28–90)	49.66 ± 17.35 (15–79)	–	–	0.002	<0.001	<0.001	0.06
UPDRS I total	–	3.84 ± 3.97 (0–12)	3.27 ± 2.99 (0–12)	–	–	0.54	<0.001	<0.001	0.16
UPDRS II total	–	14.21 ± 4.66 (3–21)	10.39 ± 4.49 (1–20)	–	–	0.004	<0.001	<0.001	0.07
UPDRS III T	–	43.68 ± 14.36 (14–62)	34.71 ± 13.34 (12–55)	–	–	0.02	<0.001	<0.001	0.67
UPDRS IV Total	–	3.26 ± 2.51 (0–9)	1.29 ± 1.69 (0–6)	–	–	0.01	<0.001	<0.001	0.04
Levodopa equivalent dose [mg]	–	882.63 ± 398.59 (0–1530)	679.15 ± 322.42 (0–1140)	–	–	0.07	<0.001	<0.001	1.00

Values are reported as mean ± standard deviation (range) or absolute and percentage frequency (%) for continuous and categorical variables, respectively. Differences between Parkinson’s disease patients and healthy controls and between Parkinson’s disease groups at baseline were assessed using ANOVA models (for continuous demographic and general clinical variables) and Chi-square test (for all categorical variables). Test for linear trend was estimated in both Parkinson’s disease groups and group-by-time interaction was assessed to evaluate longitudinal between-group differences. *P* values were adjusted for multiple comparisons at *p* < 0.05.

*DBS* deep brain stimulation, *Candidates for DBS* patients eligible for DBS, *HC* healthy controls, *Non-candidates* patients not eligible for DBS, *PD* Parkinson’s disease, *UPDRS* Unified Parkinson’s disease rating scale.

### Baseline functional brain network in Parkinson’s disease patients

#### Parkinson’s disease groups vs controls

**Fig. 2:**
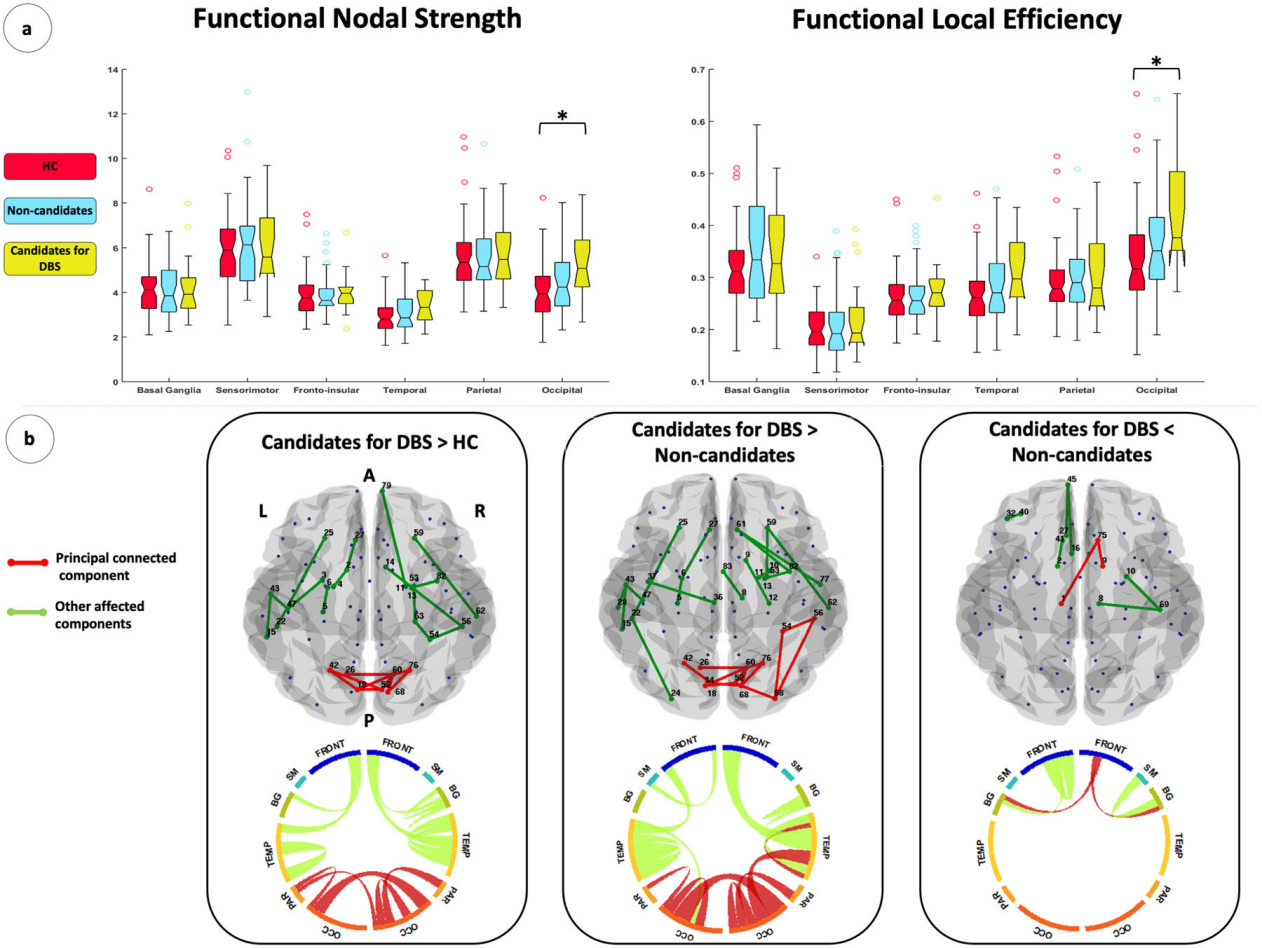
Functional connectome alterations in Parkinson’s disease patients relative to healthy controls at baseline. **a** Box plot of lobar functional nodal strength and local efficiency in healthy controls, patients eligible for DBS over time (Candidates for DBS), and patients not eligible for future DBS (Non-candidates). The black horizontal line in each box plot represents the median, the two lines just above and below the median represent the 25th and 75th percentiles, whiskers represent the minimum and maximum values, and all the dots outside the confidence interval are considered as outliers. *p < 0.05, Bonferroni-corrected for multiple comparisons. **b** Regional analysis results (NBS), from left to right: increased functional connectivity in candidates for DBS relative to controls; increased functional connectivity in Candidates for DBS relative to Non-candidates; decreased functional connectivity in Candidates for DBS relative to Non-candidates. In the upper part of the B section, abnormal functional connections are represented in a glass brain. In the lower part, lobar regions are arranged as a ring (the size of the regions are proportional to the number of the nodes included). The principal (or largest) connected components are shown in red; other connected components, not included in the principal connected components, are shown in green. Supplementary Table 2 reports affected functional connection values as well as corresponding statistical significance values. Supplementary Table 5 reports the names of each brain node with the corresponding number. A anterior, BG basal ganglia, Candidates for DBS patients eligible for DBS, FRONT frontal, HC healthy controls, L left, Non-candidates patients not candidate to DBS, OCC occipital, PAR parietal, P posterior, R right, SM sensorimotor, TEMP temporal.

Global network metrics were similar between patients eligible for DBS over the follow-up and controls (Table 2). On the other hand, lobar network analysis showed increased mean nodal strength, local efficiency, and clustering coefficient of the occipital areas in patients eligible for DBS relative to controls (Table 2 and Fig. 2a). Non-candidates for surgery were characterized by global and lobar functional architecture similar to controls (Table 2).

**Table 2:** Graph analysis properties of brain global and lobar networks in healthy controls and PD patient groups.

Regions	Graph analysis properties	HC	Candidates for DBS	Non-candidates	*p*: Non-candidates vs HC	*p*: Candidates for DBS vs HC	*p*: Candidates for DBS vs Non-candidates	*p*: for linear trend, Candidates for DBS	*p*: for linear trend, Non-candidates	*p*: for differential trend between PD groups
Global	Nodal strength	4.03 ± 0.94 (2.48–7.57)	4.36 ± 0.85 (2.68–6.40)	4.12 ± 0.86 (2.86–7.28)	1.00	0.68	0.94	0.002	0.15	0.003
	Path length	6.71 ± 1.12 (4.06–9.98)	6.32 ± 1.00 (4.33–8.35)	6.66 ± 0.97 (4.12–8.44)	1.00	0.68	0.75	0.01	1.00	0.64
	Local efficiency	0.26 ± 0.05 (0.16–0.46)	0.30 ± 0.05 (0.20–0.43)	0.28 ± 0.05 (0.20–0.42)	0.87	0.11	0.66	0.004	0.15	<0.001
	Clustering coefficient	0.16 ± 0.03 (0.09–0.30)	0.18 ± 0.03 (0.11–0.26)	0.17 ± 0.03 (0.11–0.27)	0.94	0.19	0.92	0.01	0.14	0.001
Sensorimotor	Nodal strength	5.87 ± 1.67 (2.53–10.35)	6.05 ± 1.66 (2.51–11.57)	6.19 ± 1.95 (2.59–14.33)	1.00	1.00	1.00	0.03	1.00	0.17
	Path length	5.97 ± 1.23 (3.40–9.53)	6.33 ± 2.94 (3.59–9.30)	6.04 ± 1.07 (3.01–8.77)	1.00	0.70	1.00	1.00	0.90	1.00
	Local efficiency	0.31 ± 0.08 (0.16–0.51)	0.33 ± 0.09 (0.15–0.53)	0.35 ± 0.11 (0.14–0.70)	0.16	0.99	1.00	0.052	0.76	0.34
	Clustering coefficient	0.18 ± 0.05 (0.09–0.30)	0.19 ± 0.06 (0.07–0.38)	0.20 ± 0.08 (0.07–0.50)	0.12	1.00	1.00	0.27	0.69	0.78
Frontal insular	Nodal strength	3.90 ± 0.97 (2.35–7.49)	3.96 ± 0.88 (2.22–6.37)	3.87 ± 0.89 (2.34–7.34)	1.00	1.00	1.00	0.03	0.16	0.01
	Path length	6.64 ± 1.14 (4.00–9.73)	6.41 ± 1.07 (4.67–10.30)	6.59 ± 0.99 (4.02–9.84)	1.00	1.00	1.00	0.01	1.00	0.11
	Local efficiency	0.26 ± 0.05 (0.17–0.45)	0.27 ± 0.05 (0.17–0.39)	0.26 ± 0.05 (0.17–0.46)	1.00	1.00	1.00	0.01	0.08	0.003
	Clustering coefficient	0.17 ± 0.04 (0.10–0.30)	0.17 ± 0.04 (0.10–0.26)	0.16 ± 0.04 (0.11–0.31)	1.00	1.00	1.00	0.02	0.07	0.004
Temporal	Nodal strength	2.94 ± 0.77 (1.63–5.65)	3.40 ± 0.73 (1.61–4.49)	3.05 ± 0.80 (1.72–5.92)	1.00	0.09	0.33	<0.001	0.39	0.001
	Path length	7.26 ± 1.28 (4.51–10.60)	6.47 ± 1.06 (5.49–10.94)	7.11 ± 1.13 (4.40–10.04)	1.00	0.06	0.18	0.001	1.00	0.03
	Local Efficiency	0.27 ± 0.06 (0.16–0.46)	0.30 ± 0.06 (0.15–0.43)	0.28 ± 0.07 (0.16–0.65)	0.65	0.06	0.59	0.01	0.31	0.002
	Clustering coefficient	0.18 ± 0.05 (0.10–0.34)	0.20 ± 0.04 (0.10–0.27)	0.18 ± 0.06 (0.09–0.44)	0.94	0.16	0.81	0.01	0.25	0.002
Parietal	Nodal strength	5.56 ± 1.54 (3.12–10.97)	5.80 ± 1.58 (3.31–8.86)	5.61 ± 1.48 (3.15–10.65)	1.00	1.00	1.00	0.09	0.37	0.08
	Path length	5.82 ± 1.01 (3.42–8.53)	5.70 ± 0.95 (3.69–7.79)	5.85 ± 1.00 (3.37–8.30)	1.00	1.00	1.00	0.07	1.00	0.32
	Local efficiency	0.29 ± 0.07 (0.19–0.53)	0.30 ± 0.07 (0.19–0.48)	0.29 ± 0.06 (0.17–0.50)	1.00	1.00	1.00	0.10	0.45	0.06
	Clustering coefficient	0.16 ± 0.04 (0.09–0.31)	0.17 ± 0.46 (0.10–0.27)	0.16 ± 0.04 (0.09–0.30)	1.00	1.00	1.00	0.24	0.54	0.14
Occipital	Nodal strength	3.99 ± 1.24 (1.76–8.23)	5.31 ± 1.59 (2.66–8.37)	4.48 ± 1.36 (2.23–8.94)	0.38	0.001	0.06	0.13	0.19	0.048
	Path length	6.67 ± 1.38 (3.84–11.55)	5.84 ± 0.97 (4.38–7.90)	6.38 ± 1.12 (3.70–9.15)	1.00	0.06	0.33	0.003	1.00	0.04
	Local efficiency	0.33 ± 0.09 (0.15–0.65)	0.42 ± 0.11 (0.27–0.65)	0.36 ± 0.10 (0.18–0.73)	0.48	0.001	0.06	0.67	0.24	0.04
	Clustering coefficient	0.21 ± 0.07 (0.09–0.44)	0.27 ± 0.09 (0.15–0.45)	0.23 ± 0.07 (0.10–0.50)	0.55	0.01	0.11	0.14	0.41	0.09
Basal ganglia	Nodal strength	4.13 ± 1.14 (2.10–8.61)	4.25 ± 1.37 (2.21–7.42)	4.02 ± 1.23 (2.24–8.90)	1.00	1.00	1.00	1.00	0.04	0.32
	Path length	7.00 ± 1.19 (4.12–10.08)	6.69 ± 1.09 (4.74–10.83)	7.11 ± 1.27 (4.27–9.64)	1.00	1.00	0.60	0.54	0.76	0.27
	Local efficiency	0.20 ± 0.04 (0.12–0.34)	0.20 ± 0.05 (0.13–0.41)	0.21 ± 0.06 (0.11–0.45)	1.00	0.60	1.00	1.00	0.03	0.15
	Clustering coefficient	0.10 ± 0.03 (0.05–0.17)	0.10 ± 0.03 (0.06–0.21)	0.10 ± 0.03 (0.05–0.22)	1.00	0.97	1.00	1.00	0.02	0.20

Values are reported as mean ± standard deviation (range). Global and lobar metrics were compared between groups using age- and sex-adjusted ANOVA models, followed by post-hoc pairwise comparisons, Bonferroni-corrected for multiple comparisons (*p* < 0.05). Test for linear trend was estimated in both PD-DBS and PD-noDBS groups, and group-by-time interaction was assessed to evaluate longitudinal between-group differences. Such models were adjusted for age, sex, LEDD at baseline, LEDD changes, and individual follow-up duration. *P* values were adjusted for multiple comparisons (*p* < 0.05).

*DBS* deep brain stimulation, *Candidates for DBS* patients eligible for DBS, *Non-candidates* patients not eligible for DBS, *HC* healthy controls, *PD* Parkinson’s disease.

Figure 2b and Supplementary Fig. 1 report the functional connectivity pattern that differs between each PD group and controls in Network-Based Statistics (NBS)^21^ analysis before permutation (uncorrected nonparametric test). Only the comparison between patients not eligible for DBS and controls reached the global statistical significance after permutations (*p* = 0.01, 10,000 permutations). Next, when the functional connectivity values of each connection (within both principal and secondary components) were obtained from uncorrected nonparametric test and compared using ANOVA models, a decreased connectivity between sensorimotor-basal ganglia, frontal-basal ganglia, and frontal-parietal regions and within the temporal network was found in candidates for DBS over time compared to controls (*p* values ranging from 0.01 to 0.048, Bonferroni-corrected). Furthermore, patients eligible for DBS showed an increased functional connectivity relative to controls within the occipital network and between occipital-parietal regions (principal component) and within temporal networks and frontal insular-temporal regions (secondary components) (*p* values ranging from 0.002 to 0.041, Bonferroni-corrected). Moreover, the non-candidate group revealed a widespread decreased functional connectivity compared to controls (*p* values ranging from 0.001 to 0.047, Bonferroni-corrected).

#### Patients candidate vs non-candidate for DBS

Global network analysis did not show differences between PD groups (Table 2). At the lobar network level, candidates for surgery showed a trend toward higher mean nodal strength and local efficiency of the occipital areas relative to patients who were not eligible for DBS (*p* = 0.06, Table 2 and Fig. 2a).

Figure 2b reports the functional connectivity pattern that differs between the two PD groups in NBS analysis before permutation (uncorrected nonparametric test). The comparison did not reach global statistical significance after permutations. When the functional connectivity values of each connection were obtained from nonparametric test and compared using ANOVA models, increased connectivity within an occipital network and between occipital-parietal regions (principal component) was found in patients eligible compared to those not eligible for surgery (Supplementary Table 2, *p* values ranging from <0.001 to 0.04, Bonferroni-corrected). In addition, the group of candidates for DBS showed increased functional connectivity in the temporal, occipital-temporal, and frontal insular-temporal networks (secondary components) relative to the other group of patients (Supplementary Table 2, *p* values ranging from <0.001 to 0.04, Bonferroni-corrected). On the contrary, a decreased connectivity between sensorimotor-basal ganglia and frontal insular-basal ganglia networks (principal and secondary components) was identified in patients eligible for DBS over time relative to those who were not eligible for surgery (Supplementary Table 2, *p* values ranging from <0.001 to 0.04, Bonferroni-corrected).

### Longitudinal functional brain network changes in Parkinson’s disease patients

Longitudinal global and lobar network analysis results are summarized in Table 2. Candidates for DBS showed a progressive functional alteration of the global network properties over time. At the lobar level, a progressive decreased nodal strength, local efficiency, clustering coefficient, and a longer path length were found in frontal insular and temporal regions in the group of patients eligible for DBS. Furthermore, they showed a progressive decreased nodal strength in the sensorimotor area and increased path length in occipital regions. In patients eligible for DBS, progressive increased nodal strength and local efficiency were found in basal ganglia. When longitudinal global and lobar linear trends were compared between patient groups, candidates for DBS showed a progressive reduced nodal strength, local efficiency, and clustering coefficient in the global network, frontal insular, temporal, and occipital regions relative to patients not eligible for surgery (Table 2). Moreover, a progressively increased path length was found in temporal and occipital regions in candidates for DBS relative to non-candidates.

**Fig. 3:**
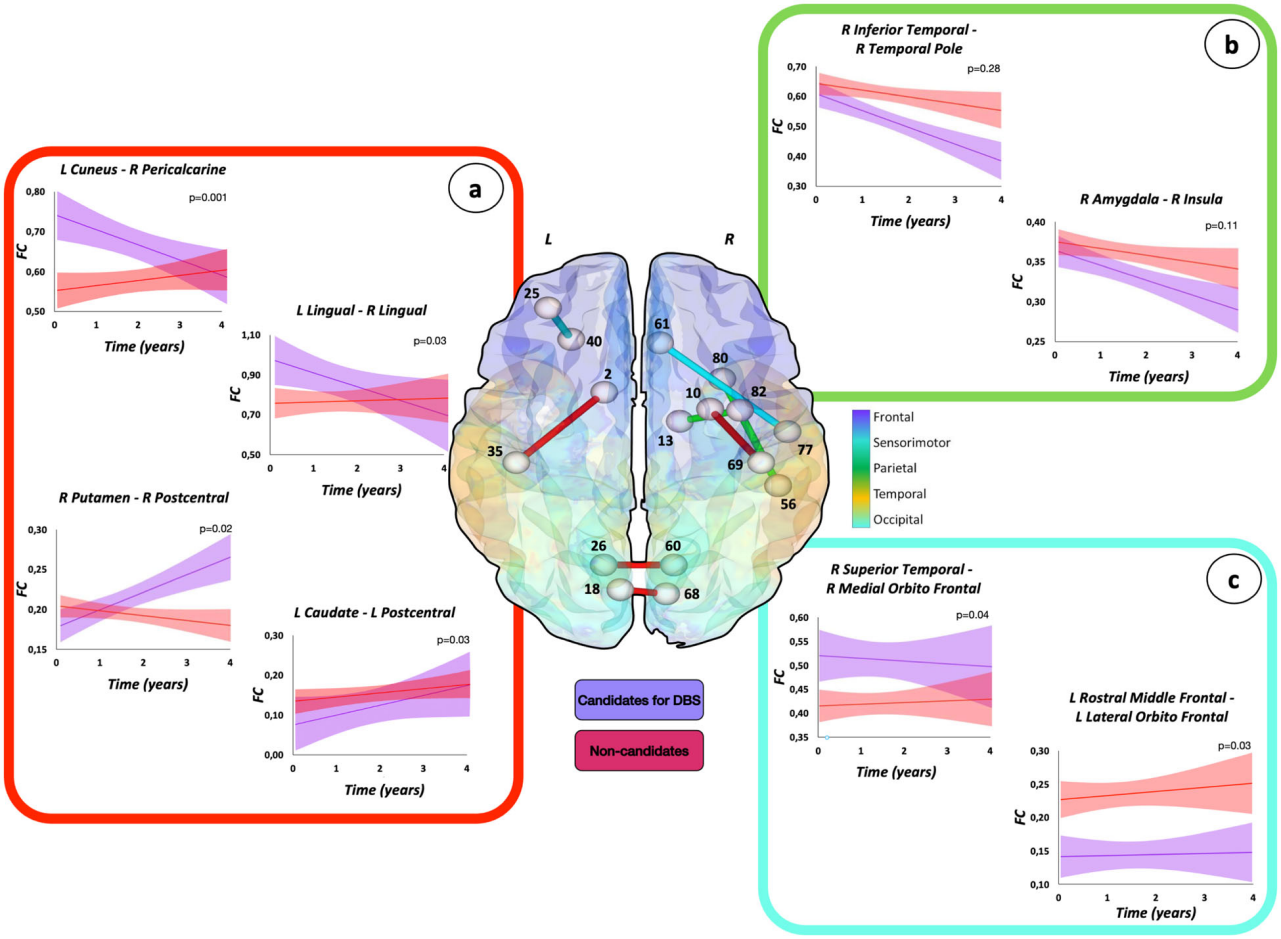
Functional connectivity changes over time in patients eligible or not to DBS. Three distinct patterns of progression are represented: **a** different trend of change between PD groups (connections in red); **b** similar trend of change (increase or decrease), with or without functional connectivity difference between the groups (connections in green); and **c** different but stable functional connectivity values over time in the two groups (connections in light blue). The effects of age, sex, levodopa equivalent daily dose at study entry, and changes of treatment over time were considered in the model. Figure reports selected findings. For further details see Supplementary Table 3. Supplementary Table 5 reports the names of each brain node with the corresponding number. Candidates for DBS patients eligible for DBS, FC functional connectivity, L left, Non-candidates patients not eligible for DBS, R right.

Functional connectivity changes over time were also compared between PD groups using NBS. Four distinct patterns of progression were identified:^9^ (1) different trends of changes between groups (increase vs decrease, increase or decrease vs stable); (2) similar trend of change (increase or decrease), with or without functional connectivity difference between the groups; (3) different but stable functional connectivity in the two groups; (4) stable functional connectivity with no difference between patient groups. When a different trend of change was observed (first condition), a decreased or stable functional connectivity within occipital and temporal networks and between occipital-parietal, occipital-temporal, temporal-parietal, and frontal insular-sensorimotor networks over time was observed in the group of patients eligible for DBS, whereas those not candidate for surgery showed increased or stable functional connectivity of these areas (Fig. 3a and Supplementary Table 3). On the contrary, candidates for DBS were characterized by increased functional connectivity involving connections from basal ganglia to occipital, temporal, sensorimotor, and frontal insular areas, where non-candidates presented stable connectivity (Fig. 3a and Supplementary Table 3). Both groups were characterized by a similar trend of change over time (second condition), with or without significant statistical difference between the two groups, within temporal, parietal, frontal insular, and sensorimotor networks and between frontal insular-temporal regions (Fig. 3b and Supplementary Table 3). Different but stable functional connectivity over time (third condition) in PD groups were found in several networks, including connections within frontal insular and temporal areas and between those regions (Fig. 3c and Supplementary Table 3).

### Correlation between functional metrics and clinical features

**Fig. 4:**
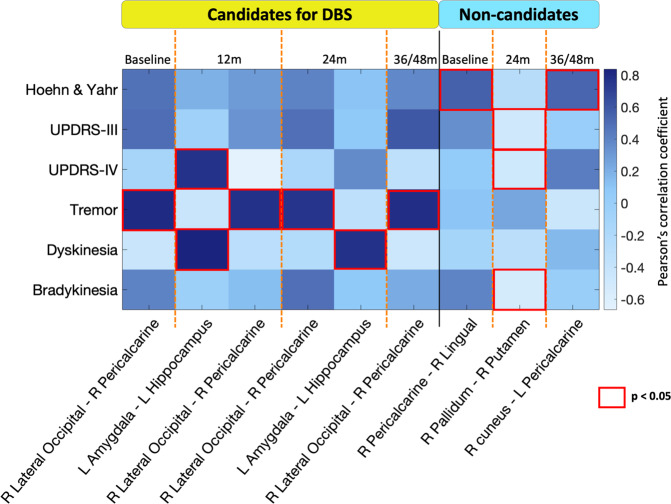
Clinical-MRI correlations between functional network properties and clinical data in each Parkinson’s disease group. Each column shows functional brain proprieties and each row clinical data at baseline and at each time point. Color scale represents Pearson’s correlation coefficient. Red squares indicate statistical significance at a threshold of *p* < 0.05. For further details see Supplementary Table 4. Candidates for DBS patients eligible for DBS, L left, m months, Non-candidates patients not eligible for DBS, R right, UPDRS Unified Parkinson’s disease rating scale.

Figure 4 and Supplementary Table 4 show the significant correlations between baseline functional network metrics and clinical variables at baseline and at each time point in PD groups.

In patients eligible for DBS over the follow-up, functional connectivity within the occipital network was related with tremor (UPDRS tremor subscore) at baseline (*r* = 0.80, *p* < 0.001), at 12 months (*r* = 0.77, *p* = 0.001), at 24 months (*r* = 0.75, *p* = 0.002) and at 36/48 months (*r* = 0.78, *p* = 0.001). Furthermore, the functional connectivity between amygdala and hippocampus was related to dyskinesia (UPDRS IV dyskinesia subscore) at 12 and 24 months (*r* = 0.84 and *r* = 0.77, *p* < 0.001, respectively) and to UPDRS IV total at 12 months (*r* = 0.76, *p* < 0.001).

In patients not eligible for DBS, functional connectivity within occipital network was related to HY score at baseline (*r* = 0.55, *p* < 0.001) and at 36/48 months from the study entry (*r* = 0.54, *p* = 0.002, respectively). Moreover, the functional connectivity within basal ganglia network was related to the following motor scales at 24 months: bradykinesia (*r* = −0.53, *p* < 0.001), UPDRS III total (*r* = −0.49, *p* = 0.002) and UPDRS IV total (*r* = −0.48, *p* = 0.003).

## Discussion

Resting-state functional connectivity is now considered a valuable measure of disease progression and provides biomarkers to monitor functional changes in PD, as well as in other neurological disorders^12^. For the first time, the present RS-fMRI study provides new insights into the dynamic evolution of brain networks in PD patients eligible or not for DBS surgery due to its longitudinal design. A major strength of the study, with regards to candidates for DBS, was the evaluation of connectivity features several years before becoming clinically eligible for DBS treatment.

At baseline, the two PD groups had similar demographic features, disease stage, and duration. However, although candidates for DBS met the criteria for neurostimulation treatment over time, their motor characteristics and brain connectivity already differed from the other group of patients at study entry. Indeed, the study revealed the following main findings at baseline: (i) hyperconnectivity of the posterior brain regions (occipital and occipital-parietal regions) as well as increased mean nodal strength and local efficiency of the occipital areas in the group of candidates for DBS relative to both healthy controls and patients not eligible for DBS; (ii) decreased connectivity between basal ganglia and both sensorimotor and frontal regions in candidates for DBS compared to non-candidates for surgery. Increased functional connectivity is a common finding in neurodegenerative diseases^22,23^, but the mechanism underlying it is still unclear. It may simply reflect the primary disease process or appear in response to altered function elsewhere in the brain (often explained as a compensation mechanism). Several authors explored basal ganglia-sensorimotor functional connectivity in PD patients reporting that levodopa medications significantly increase their connectivity;^24,25^ on the contrary, both PET and RS-fMRI studies showed that a metabolic and functional connectivity increase in the posterior areas may be a downstream effect of functional changes in the basal ganglia-sensorimotor areas^26^. We hypothesize that the baseline findings we reported may be secondary to worse response to dopaminergic therapy in candidates for DBS than in non-candidates for neurostimulation (also confirmed by more severe motor signs and symptoms in the former group despite a slightly higher LEDD) leading to basal ganglia-sensorimotor connectivity reduction as well as an increase in posterior region connections.

Over time, patients eligible for DBS were characterized by a more severe clinical motor progression than the other group of patients. In reference to fMRI analysis, PD patient groups showed different brain network changes/reorganization over the years. Our longitudinal RS-fMRI findings, in fact, reveal widespread brain network changes when the group of candidates for surgery over time became clinically eligible for DBS.

We hypothesize that the decreased functional connectivity of posterior regions over time represents a progressive loss of the initial ability to compensate for a dysfunction in the sensorimotor/frontal-basal ganglia networks that already exist at study entry in the group of patients eligible for DBS.

The progressively increased nodal strength and local efficiency in the basal ganglia region in patients not eligible for DBS and the increased functional connectivity of the basal ganglia networks in candidates for DBS relative to non-candidates could be secondary to the increased LEDD over the years in both groups. Despite the increased functional connectivity of basal ganglia was higher in the group of candidates for DBS (probably due to a greater LEDD increase), these patients showed more severe motor progression becoming thus eligible for DBS. These findings could be explained by reduced efficacy of dopaminergic medication over time and, at least partially, by the reduced connectivity in the posterior regions in patient candidate for DBS.

We suppose that considering surgery sooner during the course of the disease would prevent or delay not only motor signs and symptoms but also brain circuit alterations, being those causes or consequences of the motor impairment. Recent data, in fact, support this idea considering DBS treatment far earlier than currently applied^3,5,27^. Findings by the Earlystim study^4^ suggested that DBS at earlier stages of PD is associated with substantial clinical benefits, widening the spectrum of patients with PD eligible for such a treatment.

In patients eligible for DBS over time we found that increased functional connectivity in the occipital areas was positively related to tremor severity (UPDRS III tremor subscore) at study entry and up to 36/48 months from the baseline. In patients who were not eligible for DBS over time, the functional connectivity within basal ganglia was negatively related with bradykinesia (UPDRS III bradykinesia subscore), UPDRS III, and UPDRS IV. From the pathophysiological perspective, PD is typically attributed to the dysfunction of the basal ganglia networks, including the striatal-thalamic-cortical circuit, triggered by a deficit in dopaminergic nigrostriatal neurons^28^. However, as previously shown, this theory can explain symptoms like bradykinesia, but largely fails to explain tremor^29^. In several clinical studies, the severity of tremor is independent of the amount of dopamine deficiency^30–32^, whereas some post-mortem studies showed that PD patients with tremor have less dopaminergic dysfunction than non-tremor patients^33,34^. Our findings contribute to improving the knowledge of a complex picture revealing the potential contribution of cortical regions in tremor symptoms.

This study is not without limitations. First, the sample size is relatively small and progressively reduces over time, mainly at 36 and 48 months. This issue may explain why NBS analysis did not reach the global statistical significance after permutations in all the comparisons including candidates for DBS. Further studies with a larger patient population are needed to confirm and support our results. Secondly, PD patients were assessed in ON status. However, our analysis accounted for both LEDD at baseline and changes over time. Thirdly, we did not have longitudinal RS-fMRI data in healthy controls. Thus, we cannot ignore that part of the functional reorganization we observed in patients might be related to aging effects. Furthermore, we used a 1.5 T MRI scanner, which is characterized by a lower BOLD signal-to-noise ratio compared with higher field scanners. Moreover, patients selected as candidates for DBS did not undergo surgery at this stage. Future analyses with electrode reconstruction in the preoperative RS-fMRI space would be crucial to evaluate the relationships between the investigated functional networks and postoperative clinical outcomes. Lastly, it would be interesting to develop a connectivity machine-based learning technique that leads to a robust prediction model for better establishing which patients would be eligible for undergoing DBS according to functional connectivity features; going forward, a wider sample of PD patients would be necessary.

To conclude, this study showed different connectivity networks in PD patients eligible or not to DBS, suggesting for the first time that graph analysis and connectomics might represent a powerful approach to help clinicians establish the correct indication to DBS in PD. More precisely, specific RS-fMRI features, such as occipital hyperconnectivity and/or basal ganglia-sensorimotor/frontal hypoconnectivity together with progressively increased connectivity between basal ganglia and sensorimotor/frontal areas and decreased connectivity in the posterior regions, could be potential red flags in favor of the DBS procedure. The implementation of the current clinical criteria and the routine brain MRI with network analysis may not only support the clinical indication to DBS but could also be useful in predicting which patients would be eligible for undergoing DBS in the earlier stages of PD, thus allowing to treat patients before the occurrence of brain circuit alterations.

## Methods

### Subjects and group definition

One hundred fifty-four patients with idiopathic PD^35^ were prospectively recruited at the Clinic of Neurology, Faculty of Medicine, University of Belgrade, Serbia, within the framework of an ongoing longitudinal project. They were assessed by clinical, cognitive/behavioral, and brain MRI evaluations at study entry (baseline) and every year for a maximum of 4 years. For the purpose of the present analysis, patients were excluded if they had: disease duration less than 4 years at study entry (according to the Earlystim trial)^3,5^, severe dementia^36^, acute psychosis, or major depression with suicidal ideation^37^ (due to their absolute contraindication for DBS treatment), severe cerebrovascular disorders or intracranial masses on routine MRI, and incomplete MRI or motion artifacts during the scan. Subsequently, according to motor symptoms and signs, general clinical information, LEDD^20^, and cognitive and mood data, the PD population was divided into two groups: (1) patients eligible for DBS if they suffered from troublesome dyskinesia and/or severe motor fluctuations causing reduced quality of life despite medications adjustment, and/or refractory marked tremor over 4 years; (2) patients who did not meet the criteria to undergo DBS surgery over the follow-up. According to inclusion and exclusion criteria, 60 patients were enrolled in the study (19 candidates and 41 non-candidates for DBS over time); notably, patients selected as candidates for DBS did not undergo surgery at this stage. The outline of the patient selection process is summarized in Fig. 1 and Supplementary Fig. 2. Sixty age- and sex-matched healthy controls without any neurological and psychiatric disorders were also recruited and underwent clinical, cognitive, and MRI assessments at study entry.

The study received approval from the ethics committee on human experimentation of the Faculty of Medicine—University of Belgrade (No. 175090). Written informed consent was obtained from all patients participating in the study.

### Clinical evaluation

At study entry and at each follow-up visit, an experienced neurologist blinded to MRI results performed clinical assessments. Patients were examined in ON state (i.e., period when the dopaminergic medication is working and symptoms are well controlled). Demographic, general clinical, and family data (age, sex, education, handedness, age at onset, site of onset, PD duration, medications, and family history) were obtained using a semi-structured interview. LEDD was calculated^20^. Disease severity was defined using the HY stage score^18^. The UPDRS^19^ was used to evaluate non-motor symptoms (UPDRS I), motor symptoms (UPDRS II), motor signs (UPDRS III), and motor complications (UPDRS IV). UPDRS III rigidity, tremor, and bradykinesia sub-scores were also calculated. The presence of dyskinesia and motor fluctuations was evaluated according to the UPDRS IV sub-scores.

### Neuropsychological and behavioral evaluations

Expert neuropsychologists, blinded to clinical and MRI results, performed neuropsychological and behavioral evaluations at each visit in both PD patients and healthy controls. None of the patients received cholinesterase inhibitors at study entry or during the follow-up. The following tests were administered: the Addenbrooke’s Cognitive Examination-revised (ACE-R);^38^ the Rey Auditory Verbal Learning Test^39^, pattern and spatial recognition memory tests from the Cambridge Neuropsychological Test Automated Battery (CANTAB);^40^ executive functions with the digit span backward, Intra/Extra-Dimensional Set Shift test from the CANTAB^40^, and the Stroop color-word test; the digit ordering test and the letter cancellation test (attention and working memory);^41^ the Boston Naming Test^42^ and the language subtest of ACE-R; semantic^42^ and phonemic fluencies;^43^ the Hooper Visual Organization test^44^ and the visuospatial subtest of ACE-R (visuospatial abilities). Patients were defined as having severe dementia in accordance with MDS-Task Force criteria^45^. The mood was evaluated with the Hamilton Depression Rating Scale score^37^, Hamilton Anxiety Rating scale score^46^, and Apathy Evaluation Scale^47^.

### MRI acquisition

At each visit, brain MRI scans were obtained for both PD patients and healthy controls on the same 1.5 Tesla Philips Achieva system machine (Philips Medical Systems, Best, the Netherlands). Patients were scanned 90–120 min after their regular morning dopaminergic therapy administration (ON state). The following MRI sequences were obtained: (i) Dual-Echo Turbo Spin-Echo (repetition time [TR] = 3125 ms, echo time [TEs] = 20/100 ms, echo train length [ETL] = 6,44 axial slices, thickness = 3.0 mm, matrix size = 256 × 247, field of view [FOV] = 240 × 232 mm^2^; voxel size = 0.94 × 0.94 × 3 mm, in-plane sensitivity encoding [SENSE] parallel reduction factor, 1.5), (ii) three-dimensional (3D) sagittal T1-weighted Turbo-Field-Echo (TR = 7.1 ms, TE = 3.3 ms, inversion time = 1000 ms, flip angle = 8°, matrix size = 256 × 256 × 180, FOV = 256 × 256 mm^2^, section thickness = 1 mm, voxel size = 1 × 1 × 1 mm), and (iii) gradient-echo echo planar imaging for RS-fMRI (TR = 3000 ms, TE = 35 ms, flip angle = 90°, matrix size = 128 × 128, FOV = 240 × 240 mm^2^, voxel size = 1.88 × 1.88 × 4 mm, slice thickness = 4 mm, 200 sets of 30 contiguous axial slices) sequences were acquired and subsequently analyzed for the purpose of this study. For the latter sequence, participants were instructed to remain motionless, to keep their eyes closed, and to try thinking of nothing.

### MRI analysis

**Fig. 5:**
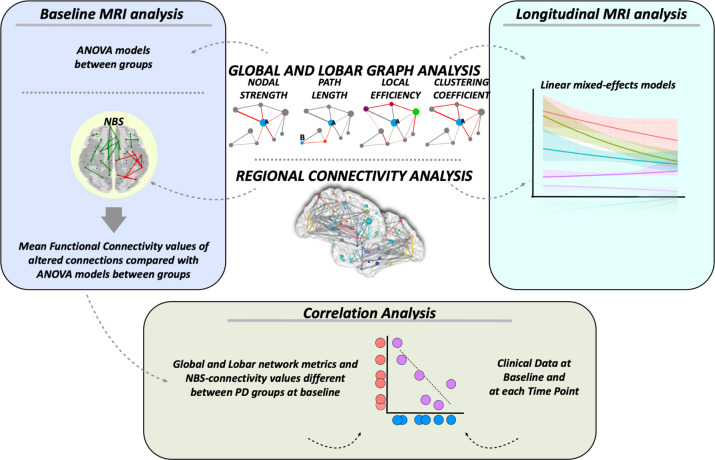
MRI processing pipeline. Baseline MRI analysis. Global and lobar network topological metrics were compared between groups using ANOVA models. NBS analysis compared functional connectivity between groups. Mean functional connectivity values of the resulting altered connections were obtained and compared between groups using ANOVA models. Longitudinal MRI analysis. Linear mixed-effects models were implemented to investigate global and lobar network measures and functional connectivity changes over time. Correlation analysis. Partial correlations were assessed between baseline fMRI metrics (global and lobar network metrics and NBS-connectivity values, which were found to be different between PD groups) and clinical data at baseline and at each time point using Pearson’s correlation coefficient. NBS network-based statistics.

MRI analysis was performed at the Neuroimaging Research Unit, IRCCS Ospedale San Raffaele, Milan, Italy, by two experienced observers, blinded to subjects’ identity and diagnosis. A methodological framework for the MRI analysis is summarized in Fig. 5.

#### Brain parcellation

T1-weighted images were processed and parcellated using the Freesurfer suite (V 5.3 http://surfer.nmr.mgh.harvard.edu/), resulting in 83 areas (Supplementary Table 5), which were used to define brain nodes for the network analysis. Briefly, images were automatically processed following the standard free surfer procedure, which includes brain extraction, segmentation of gray matter (GM) and white matter (WM), and parcellation in cortical and subcortical regions. GM was parcellated according to a standardized atlas^48^ and segmented in 68 cortical areas. Segmentations of bilateral thalamus, caudate, putamen, globus pallidus, amygdala, hippocampus, and brainstem were obtained from the Freesurfer pipeline and added to the previous 68 areas.

#### RS-fMRI preprocessing

RS-fMRI data processing was carried out using the FMRIB software library (FSLv5.0). First, T1-weighted images were skull stripped using the Brain Extraction Tool and segmented in GM, WM, and cerebrospinal fluid (CSF) maps using the FMRIB’s Automated Segmentation Tool. The resulting images were registered into the RS-fMRI native space of each subject through a seven degree-of-freedom (DOF) linear affine transformation using FMRIB’s Linear Image Registration Tool. The first four volumes of the fMRI data were removed to reach complete magnet signal stabilization. Then, individual RS-fMRI images were processed using MELODIC (Multivariate Exploratory Linear Optimized Decomposition into Independent Components; version 3.10; http://www.fmrib.ox.ac.uk/fsl/melodic/)^49^. The following FSL-standard preprocessing pipeline was applied: (1) motion correction using MCFLIRT; (2) high-pass temporal filtering (lower frequency: 0.01 Hz); (3) spatial smoothing (Gaussian Kernel of FWHM 6 mm); (4) single-session independent component analysis (ICA). The resulting independent components (ICs) were inspected visually by analyzing their spatial patterns and temporal characteristics^49^, and those ICs that could be attributed to subject head movement, physiological noise, or CSF fluctuations were removed from the original settings using the “fslregfilt” tool. Head motion parameters (mean absolute cumulative translation and mean rotation) are reported in Supplementary Table 6. Differences between PD patients and healthy controls and between PD groups at baseline were assessed using one-way ANOVA. Linear regression models were built to assess longitudinal head motion parameter changes. Head motion parameters were considered as the dependent variable in each model, which also included individual follow-up duration as covariate (independent variable). Baseline and longitudinal analyses reported no significant differences in the amount of head movement in PD subgroups and controls (Supplementary Table 6).

#### Functional connectome reconstruction

Functional connectivity matrices were obtained based on correlation analysis. Meantime series were extracted from each region of interest by averaging the signal from all voxels within each region. RS-fMRI data were masked with the subject’s GM map in order to consider only voxels corresponding to GM and avoid the effect of atrophy. Cortical GM was segmented using SPM12, while basal ganglia (i.e., bilateral caudate, globus pallidus, putamen, and thalamus), hippocampus, and amygdala maps were obtained using FIRST in FSL. The Pearson’s correlation coefficient between the mean time-series of each node pair, indicating the level of functional connectivity between regions *i* and *j*, was entered into cell *c*(*i*,*j*) of the matrix. Pearson’s correlation coefficients were then converted to *z*-scores using Fisher’s r-to-z transformation. Negative values were set as “NaN” to mark these brain regions as unconnected^50^. Functional connections of each subject were required to be present in each structural connectivity matrix, i.e., we measured functional interactions only where an anatomical connection between two areas occurs in the independent healthy control sample. The following paragraph describes the construction of the structural network in the independent healthy control group.

#### Construction of the white matter network in the independent healthy controls

MRI scans of an independent healthy control group (*N* = 99, mean age = 51.22 ± 13.90, sex = 61 female and 38 male, mean MMSE = 29.72 ± 0.63) were obtained using the same MRI scanner as controls and patients of the present study, and included: (i) DE turbo SE; (ii) 3D sagittal T1-weighted fast field echo; and (iii) pulsed gradient SE single-shot echo-planar (TR = 6713 ms, TE = 86 ms, flip angle = 90°, matrix size = 112 × 110, FOV = 224 × 220 mm; 50 contiguous, 2.6-mm thick, axial slices, voxel size = 2 × 2 × 2.6 mm, SENSE Parallel Reduction Factor In-Plane = 2), with diffusion-encoding gradients applied in 65 non-collinear directions (b factor = 1000 s/mm^2^) and seven averages of the b = 0 acquisition. Brain parcellation was performed as described above. The diffusion-weighted data were skull stripped by using the Brain Extraction Tool implemented in FSL. Diffusion-weighted (DW) images were corrected for distortions caused by eddy currents and movement by using an implementation of a previously described algorithm described in ref. ^51^ (http://white.stanford.edu/newlm/index.php/DTI_Preprocessing#dtiRaw_Preprocessing_Pipeline). This eddy current and motion correction step combines rigid body three-dimensional motion correction (six parameters) with constrained nonlinear warping (eight parameters) based on a model of the expected eddy current distortions. Previous transformations were concatenated to a further affine transformation calculated to register DW images onto the MNI space and were applied to DW data. The diffusion tensor (DT) was estimated on a voxel-by-voxel basis by using the DTIfit toolbox, which is part of the FMRIB Diffusion Toolbox within FSL to obtain fractional anisotropy (FA) maps. WM fiber tracts were reconstructed with Diffusion Toolkit/Trackvis (https://www.nitrc.org/projects/trackvis) using the Fiber Assignment by Continuous Tracking (FACT) algorithm^50,52^. A whole-brain tractogram was obtained by initiating tractography in each WM voxel of the brain, following the principal diffusion direction. Fiber tracking was stopped if the reconstructed fiber entered a voxel with FA <0.15, if the streamline made a turn with a curvature angle of more than 45°, or when the trajectory of the traced fiber exceeded the brain mask. An individual brain network was obtained for each healthy control by using the following procedure^52,53^. First, the streamlines from the whole-brain tractogram touching each couple i and j of nodes were selected. Then, the number of streamlines for each of these tracts was calculated and inserted in an adjacency matrix. If there was no streamline connecting a couple of nodes, then a zero was inserted in the corresponding cell to describe the lack of connections between those nodes. To avoid the presence of spurious fibers, we set to zero all connections with fewer than three fibers. Moreover, to avoid considering spurious connections in our analysis, we set to zero the connections that were present in less than 40% of the independent healthy control sample^52,54^. This procedure was repeated for each i and j couple of nodes, resulting in a connected undirected weighted matrix. Functional connectome matrices are dense and we need to apply a threshold in order to avoid spurious functional connections. Up to date, there is no standard technique to threshold connectivity matrices. In order to be as conservative as possible, we decided to study functional connectivity only where an anatomical connection is present masking functional matrix with a structural one. Finally, we masked the functional matrices of patients and controls included in our study using the structural matrix obtained from the independent healthy control sample. If the anatomical connection was not present in the structural matrix, we set to ‘not a number’ the corresponding correlation coefficient in the functional matrix of patients and controls.

### Statistical analysis

#### Demographic, clinical, and cognitive data

Demographic, clinical (motor and non-motor), and cognitive data at baseline were compared between groups using ANOVA models or Chi-square test at study entry. Test for linear trend was estimated in both PD groups and group-by-time interaction was assessed to evaluate longitudinal between-group differences. *P* values were Bonferroni-corrected for multiple comparisons at *p* < 0.05. All statistical analyses were performed using R Statistical Software (version 4.0.3; R Foundation for Statistical Computing, Vienna, Austria).

#### Baseline MRI analysis

Global and lobar metrics were compared between groups using age- and sex-adjusted ANOVA models, followed by post-hoc pairwise comparisons, Bonferroni-corrected for multiple comparisons (*p* < 0.05, R Statistical Software).

NBS compared functional connectivity between groups adjusting for age and sex. NBS is a nonparametric test and has the potential of identifying any connected component and a network comprising functional connectivity differences between groups. Per each between-group comparison, the test statistic is computed for each functional connection, obtaining corresponding *p* values (one *p* value per connection). Connections presenting a *p* value <0.05 are considered to be part of the altered functional connectivity pattern. The principal (or largest) connected component and the smaller clusters of altered connections (secondary), which are not included in the principal connected component, are then identified (red and green connections, respectively, in Fig. 2b and Supplementary Fig. 1). At this point, since the inherent massive number of multiple comparisons that must be performed and the great effort in testing the normality per each connection, the permutation test is used to ascribe a *p* value controlled for the family-wise error to the considered altered functional connectivity pattern. The permutation test re-samples *N* times the total number of observations in the subject group to build an empirical estimate of the null distribution from which the test statistic has been drawn. The p values obtained after 10,000 permutations are reported in the result section for each comparison (i.e., the global significance of the altered functional connectivity pattern). Then, regardless of global statistical significance after permutations, the mean functional connectivity values of the resulting altered connections (in both the principal and secondary components) were obtained and compared between groups using age- and sex-adjusted ANOVA models, followed by post hoc pairwise comparisons, Bonferroni-corrected for multiple comparisons (*p* < 0.05, R Statistical Software).

#### Longitudinal MRI analysis

Changes over time of the functional network metrics were assessed with general linear models using time as a continuous variable. Test for linear trend was estimated in both PD patient groups, and group-by-time interaction was assessed to evaluate longitudinal between-group differences. Such models were adjusted for age, sex, LEDD at baseline, LEDD changes, and individual follow-up duration. *P* values were Bonferroni-corrected for multiple comparisons at *p* < 0.05.

Concerning regional connectivity analysis, linear mixed-effects models were implemented in NBS to investigate functional connectivity changes over time at *p* < 0.05, Bonferroni-corrected for multiple comparisons. The effects of age, sex, LEDD at baseline, LEDD changes, and individual follow-up duration were considered in the regression models.

#### Correlation analysis

Partial correlations were assessed between baseline fMRI metrics (global and lobar network metrics and NBS-connectivity values, which were found to be different between PD groups) and baseline clinical scales using Pearson’s correlation coefficient (*p* < 0.05). Analysis was adjusted for age, sex, and LEDD at baseline. Furthermore, correlations between fMRI measures at baseline and clinical scales at each time point (12, 24, 36/48 months) were also explored using Pearson’s correlation coefficient (*p* < 0.05), to evaluate the possible effect of brain functional network alterations on clinical progression in PD groups. Analysis was adjusted for age and sex, LEDD at baseline, and LEDD changes. Correlation analyses were performed using Matlab, controlling for multiple comparisons using Bonferroni adjustment.

### Reporting summary

Further information on research design is available in the Nature Research Reporting Summary linked to this article.

## Supplementary information

Supplementary Information

Reporting Summary

## Data Availability

The dataset used and analyzed during the current study will be made available by the corresponding author upon request to qualified researchers (i.e., affiliated to a university or research institution/hospital).
