# Supplementary material for: Functional connectivity in Parkinson’s disease candidates for deep brain stimulation
Source: NPJ Parkinsons Dis. 2022 Jan 10;8:4. doi: 10.1038/s41531-021-00268-6 (PMC8748462; doi:10.1038/s41531-021-00268-6)
Supplement: Supplementary file 1 — Supplementary Information [file 41531_2021_268_MOESM1_ESM.pdf]

Supplementary Table 1: Cognitive and behavioral characteristics of Parkinson's disease patients and healthy controls at the study entry and their changes over time.

Variables	HC	Candidates for DBS	Non-candidates	p: Candidates for DBS vs HC	p: Non-candidates vs HC	p: Candidates for DBS vs Non-candidates	p: for linear trend Candidates for DBS	p: for linear trend Non-candidates	p: for differential trend Candidates for DBS vs Non-candidates
**Global cognition**									
ACE-R total	96.28±3.00 (87.00-100.00)	87.42±8.22 (69.00-99.00)	89.37±7.42 (65.00-99.00)	< 0.001	< 0.001	0.37	< 0.001	<0.01	0.13
**Memory**									
RAVLT immediate recall	45.36±10.24 (21.00-65.00)	37.89±10.50 (24.00-59.00)	38.22±11.31 (17-66.00)	0.048	0.004	1.00	0.01	1.00	0.11
RAVLT delayed recall	8.92±2.74 (4.00-13.00)	12.53±2.56 (6.00-15.00)	12.37±3.16 (0.00-15.00)	0.044	0.003	1.00	0.01	0.62	0.31
PRM [% correct]	79.69±11.46 (37.50-95.83)	74.94±11.26 (50.00-96.00)	74.48±18.24 (0.00-100.00)	1.00	0.26	1.00	1.00	1.00	1.00
SRM [% correct]	73.88±10.52 (55.00-95.00)	67.63±13.47 (40.00-90.00)	70.12±16.18 (0.00-85.00)	0.35	0.49	1.00	0.001	0.81	0.73
**Language**									
BNT total	58.22±1.63 (53.00-60.00)	54.26±5.08 (42.00-59.00)	55.44±8.97 (5.00-60.00)	0.048	0.08	1.00	0.29	0.66	0.33
ACE-R language	25.90±0.30 (25.00-26.00)	22.74±2.62 (18.00-27.00)	24.15±2.57 (14.00-26.00)	< 0.001	< 0.001	0.01	1.00	1.00	1.00
**Executive functions**									
Digit span backward	6.80±1.95 (3.00-11.00)	5.78±3.02 (2.00-11.00)	5.54±1.96 (0.00-11.00)	0.04	0.12	1.00	0.001	0.004	0.31

Stroop interference [total correct]	38.24±10.34 (17.00-60.00)	34.53±12.41 (14.00-55.00)	32.61±11.60 (13.00-68.00)	0.42	0.23	1.00	< 0.001	< 0.001	0.06
IED [total errors]	41.64±28.99 (7.00-146.00)	53.26±31.70 (0.00-161.00)	59.32±38.95 (11.00-225.00)	0.69	0.46	1.00	< 0.001	0.01	0.33
**Attention**									
Digit ordering [max. span]	5.77±1.24 (4.00-8.00)	5.02±1.02 (3.00-6.50)	5.23±1.13 (3.50-8.50)	0.04	0.13	1.00	0.01	0.01	0.35
Letter cancelation correct	28.44±5.53 (20.00-41.00)	23.58±6.65 (15.00-37.00)	22.43±5.70 (0.00-39.00)	0.04	<0.001	1.00	< 0.001	<0.001	0.004
**Visuospatial abilities**									
Hooper	23.12±3.34 (13.00-30.00)	18.95±6.24 (9.50-29.00)	19.17±5.69 (9.00-30.00)	0.003	0.001	1.00	0.01	0.10	0.09
ACE-R visuospatial	15.80±0.40 (15.00-16.00)	15.68±0.58 (14.00-16.00)	15.22±1.12 (12.00-16.00)	1.00	0.40	0.18	0.01	< 0.001	0.24
**Mood/behavior**									
HDRS	2.30±3.63 (0.00-15.00)	6.42±5.77 (0.00-20.00)	4.88±5.05 (0.00-18.00)	0.01	0.03	1.00	< 0.001	< 0.001	0.98
HAMA	2.92±3.06 (0.00-11.00)	6.05±4.89 (0.00-16.00)	5.17±5.75 (0.00-21.00)	0.03	0.08	1.00	0.01	< 0.001	0.99
Apathy scale	1.74±2.90 (0.00-11.00)	11.26±9.93 (0.00-28.00)	10.15±7.68 (0.00-28.00)	< 0.001	< 0.001	1.00	< 0.001	0.003	0.51

Values are reported as mean ± standard deviation (range). Differences between Parkinson's disease patients and healthy controls and between Parkinson's disease groups at baseline were assessed using ANOVA models. Test for linear trend was estimated in both Parkinson's disease groups and group-by-time interaction was assessed to evaluate longitudinal between-group differences. P values were adjusted for multiple comparisons at $p < 0.05$. Abbreviations: ACE-R=Addenbrooke's Cognitive Examination-Revised; BNT=Boston Naming Test; DBS=Deep Brain Stimulation; Candidates for DBS=Patients eligible for DBS; HAMA=Hamilton Anxiety Rating Scale; HC=healthy controls; HDRS=Hamilton Depression Rating Scale; IED=Intra and Extra-Dimensional shifting; Non-candidates=Patients not eligible for DBS; PD=Parkinson's disease; PRM=Pattern Recognition Memory; RAVLT=Rey Auditory Verbal Learning Test; SRM=Spatial Recognition Memory.

Supplementary Table 2: Affected functional connection values obtained from regional analysis at baseline in patients eligible or not for DBS.

**Candidates for DBS > Non-candidates**			
**Connections**	**FC Candidates for DBS**	**FC Non-candidates**	**P value**
L amygdala - L hippocampus	0.47 ± 0.23 (0.23-1.16)	0.33 ± 0.24 (0.20-0.96)	0.04
R insula - R amygdala	0.34 ± 0.17 (0.13-0.62)	0.23± 0.17 (0.01-0.59)	0.03
L superior temporal - L bankssts	0.70 ± 0.29 (0.18-1.31)	0.50 ± 0.26 (0.04-1.20)	0.01
L pericalcarine - L cuneus	0.71 ± 0.26 (0.31-1.13)	0.54 ± 0.25 (0.09-1.05)	0.02
L superior parietal - L cuneus	0.66 ± 0.31 (0.18-1.34)	0.48 ± 0.21 (0.13-0.97)	0.01
R lingual - L cuneus	0.77 ± 0.29 (0.16-1.18)	0.58 ± 0.25 (0.04-1.09)	0.01
R pericalcarine - L cuneus	0.72 ± 0.23 (0.21-1.09)	0.53 ± 0.25 (0.11-1.11)	0.01
L lateral occipital - L inferior temporal	0.43 ± 0.26 (0.03-0.91)	0.29 ± 0.20 (0.01-0.70)	0.03
L amygdala - L medial orbitofrontal	0.31 ± 0.20 (0.10-0.82)	0.18 ± 0.14 (0.19-0.64)	0.01
L superior temporal - L middle temporal	0.62 ± 0.25 (0.17-1.10)	0.48 ± 0.23 (0.09-0.87)	0.03
R cuneus - L pericalcarine	0.63 ± 0.30 (0.07-1.17)	0.47 ± 0.23 (0.03-0.93)	0.03
L precentral - L posterior cingulate	0.42 ± 0.23 (0.13-1.06)	0.27 ± 0.19 (0.01-0.89)	0.02
L transverse temporal - L superior temporal	0.80 ± 0.37 (0.17-1.78)	0.54 ± 0.31 (0.04-1.45)	0.01
R lingual - R cuneus	0.82 ± 0.27 (0.27-1.19)	0.49 ± 0.25 (0.01-1.19)	<0.001
R pericalcarine - R cuneus	0.74 ± 0.28 (0.06-1.13)	0.53 ± 0.24 (0.01-1.10)	0.005
R lateral orbitofrontal - R entorhinal	0.38 ± 0.27 (0.01-0.81)	0.25 ± 0.14 (0.02-0.63)	0.04
R pallidum R putamen	0.44 ± 0.23 (0.23-1.16)	0.32 ± 0.24 (0.20-0.96)	0.04
R inferior temporal - R fusiform	1.13 ± 0.38 (0.43-1.83)	0.89 ± 0.34 (0.04-1.83)	0.01
R lateral occipital - R inferior temporal	0.42 ± 0.18 (0.09-0.75)	0.25 ± 0.14 (0.03-0.66)	0.001
R pericalcarine - R lateral occipital	0.52 ± 0.22 (0.17-1.07)	0.41 ± 0.21 (0.02-0.80)	0.04
R middle temporal - R lateral orbitofrontal	0.77 ± 0.33 (0.17-1.31)	0.54 ± 0.26 (0.07-1.14)	0.01
R superior parietal - R lingual	0.61 ± 0.20 (0.28-0.88)	0.39 ± 0.30 (0.03-1.43)	0.01
R insula - R medial orbitofrontal	0.31 ± 0.16 (0.06-0.69)	0.10 ± 0.12 (0.01-0.43)	0.01

R superior parietal - R pericalcarine	0.50 ± 0.25 (0.11-0.93)	0.31 ± 0.21 (0.01-0.82)	0.005
**Candidates for DBS < Non-candidates**			
**Connections**	**FC Candidates for DBS**	**FC Non-candidates**	**P value**
R superior frontal - L thalamus	0.18 ± 0.12 (0.01-0.30)	0.09 ± 0.09 (0.02-0.58)	0.04
L superior frontal - L caudate	0.21 ± 0.13 (0.01-0.30)	0.07 ± 0.10 (0.02-0.48)	0.01
R postcentral - R thalamus	0.21 ± 0.11 (0.03-0.13)	0.08 ± 0.04 (0.07-0.49)	0.03
R superior frontal - R caudate	0.23 ± 0.13 (0.01-0.28)	0.08 ± 0.06 (0.03-0.49)	0.003
R postcentral - R putamen	0.31 ± 0.09 (0.01-0.35)	0.15 ± 0.10 (0.19-0.50)	< 0.001
L frontal pole - L caudal anterior cingulate cortex	0.18 ± 0.13 (0.01-0.10)	0.05 ± 0.03 (0.02-0.43)	0.04
L frontal pole - L medial orbitofrontal	0.37 ± 0.18 (0.04-0.64)	0.24 ± 0.18 (0.05-0.75)	0.02
L pars orbitalis - rostral middle frontal	0.39 ± 0.25 (0.01-0.44)	0.22 ± 0.11 (0.01-0.99)	0.01

Values are reported as mean ± standard deviation (range). Differences between PD subgroups were assessed using age- and sex–adjusted ANOVA models, followed by post-hoc pairwise comparisons, Bonferroni-corrected for multiple comparisons ($p < 0.05$). Abbreviations: DBS=Deep Brain Stimulation; Candidates for DBS=Patients eligible for DBS; FC=Functional Connectivity; L=Left; Non-candidates=Patients not eligible for DBS; PD=Parkinson's Disease; R=Right.

Supplementary Table 3: Functional connectivity changes over time in patients eligible or not for DBS.

Pattern of progression	Lobes	Node 1	Node 2
**1) Different trend of changes between subtypes**			
**Non-candidates → Candidates for DBS ↓**	Occipital-Occipital	R cuneus	R lingual
		L lingual	R lingual
		L cuneus	L lingual
		L lingual	L pericalcarine
		R lingual	L cuneus
		L cuneus	R pericalcarine
		R cuneus	R pericalcarine
		R lateral occipital	R pericalcarine
		R lingual	R pericalcarine
	Parietal-Occipital	L superior parietal	L lateral occipital
		L superior parietal	L cuneus
		L superior parietal	R lateral occipital
		L superior parietal	L pericalcarine
		R precuneus	R lingual
		R superior parietal	L lateral occipital
		R superior parietal	R cuneus
		R superior parietal	R lingual

	Temporal-Parietal	L superior temporal	R hippocampus
		L isthmus	L superior parietal
		R isthmus	R superior parietal
	Temporal-Occipital	R fusiform	R lateral occipital
	Temporal-Temporal	L superior temporal	L bankssts
		L middle temporal	L superior temporal
		R fusiform	R inferior temporal
	Basal ganglia-Temporal	L pallidum	L entorhinal
	Basal ganglia-Basal ganglia	L pallidum	L caudate
		L pallidum	L accumbens
	Frontal insular-Frontal insular	L superior frontal	L caudal middle frontal
	Frontal insular-Sensorimotor	L superior frontal	L precentral
		R superior frontal	R precentral
	Parietal-Frontal insular	L posterior cingulate	L superior frontal
**Non-candidates → Candidates for DBS ↑**	Basal ganglia-Sensorimotor	R caudate	R postcentral
		R putamen	R postcentral
		L caudate	L postcentral
	Basal ganglia-Frontal insular	L caudate	L superior frontal
		R accumbens	R insula
	Frontal insular-Frontal insular	R lateral orbitofrontal	R pars triangularis
	Basal ganglia-Occipital	R putamen	R lateral occipital

	Basal ganglia-Temporal	L thalamus	L hippocampus
**Non-candidates ↑ Candidates for DBS ↓**	Occipital-Temporal	L lateral occipital	R inferior temporal
	Occipital-Parietal	L cuneus	R superior parietal
	Frontal insular-Frontal insular	L pars opercularis	L rostral middle frontal
**Non-candidates ↑ Candidates for DBS →**	Occipital-Temporal	R lateral occipital	R middle temporal
	Temporal-Temporal	L middle temporal	R inferior temporal
		R hippocampus	R fusiform
	Temporal-Parietal	R bankssts	R inferior parietal
	Frontal insular-Frontal insular	L rostral middle frontal	R caudal anterior cingulate
		L superior frontal	R rostral middle frontal
	Parietal-Parietal	L posterior cingulate	R posterior cingulate
**2) Similar trend of changes (increase or decrease). with or without difference between the groups**			
**Non-candidates ↓ Candidates for DBS ↓**	Occipital-Parietal	L lingual	L precuneus
	Parietal-Parietal	R precuneus	L precuneus
	Temporal-Frontal insular	R amygdala	R insula
	Temporal-Temporal	R inferior temporal	R temporal pole
		L isthmus	R isthmus
	Frontal insular-Frontal insular	L superior frontal	R superior frontal
		L rostral middle frontal	R rostral middle frontal
	Sensorimotor-Sensorimotor	L precentral	R precentral
		L post central	R precentral

**Non-candidates ↑ Candidates for DBS ↑**	Temporal-Frontal insular	R temporal pole	R insula
	Basal ganglia-Temporal	R thalamus	R entorhinal
**3) Different but stable functional connectivity values over time in the two subtypes**			
**Non-candidates → Candidates for DBS →**	Frontal insular-Temporal	R rostral middle frontal	R superior temporal
		R medial orbitofrontal	R superior temporal
		L medial orbitofrontal	L amygdala
		L insula	L entorhinal
	Frontal insular-Frontal insular	L frontal pole	L caudal anterior cingulate
		L pars orbitalis	L rostral middle frontal
		L lateral orbitofrontal	L rostral middle frontal
		R medial orbitofrontal	R insula
	Parietal-Temporal	R superior parietal	R amygdala
	Temporal-Temporal	L superior temporal	L transverse temporal
		L bankssts	L middle temporal

Legend: →: stable functional connectivity overtime; ↑: increased functional connectivity overtime; ↓: decreased functional connectivity overtime. Linear mixed-effects models were implemented in NBS to investigate functional connectivity changes over time at $p < 0.05$, Bonferroni-corrected for multiple comparisons. The effects of age, sex, LEDD at baseline, LEDD changes and individual follow-up duration were considered in the regression models. Abbreviations: DBS=Deep Brain Stimulation; Candidates for DBS=Patients eligible for DBS; L=Left; NBS=Network-based Statistics; Non-candidates=Patients not eligible for DBS; PD=Parkinson's disease; R=Right.

Supplementary Table 4: Significant correlations between functional network measures at baseline and clinical data at study entry and at each follow-up in patients eligible or not to DBS.

**fMRI measure**	**Candidates for DBS clinical data (baseline)**	**r**	**p**
R lateral occipital - R pericalcarine	Tremor	0.80	< 0.001
**fMRI measure**	**Non-candidates clinical data (baseline)**	**r**	**p**
R pericalcarine - R lingual	HY	0.55	< 0.001
**fMRI measure**	**Candidates for DBS clinical data (12 M)**	**r**	**p**
L amygdala - L hippocampus	Dyskinesia	0.84	< 0.001
L amygdala - L hippocampus	UPDRS IV	0.76	< 0.001
R lateral occipital - R pericalcarine	Tremor	0.77	0.001
**fMRI measure**	**Non-candidates clinical data (12 M)**	**r**	**p**
-	-	-	-
**fMRI measure**	**Candidates for DBS clinical data (24 M)**	**r**	**p**
L amygdala - L hippocampus	Dyskinesia	0.77	< 0.001
R lateral occipital - R pericalcarine	Tremor	0.75	0.002
**fMRI measure**	**Non-candidates clinical data (24 M)**	**r**	**p**

R pallidum - R putamen	Bradykinesia	-0.53	< 0.001
R pallidum - R putamen	UPDRS III	-0.49	0.002
R pallidum - R putamen	UPDRS IV	-0.48	0.003
**fMRI measure**	**Candidates for DBS clinical data (36-48 M)**	**r**	**p**
R lateral occipital - R pericalcarine	Tremor	0.78	0.001
**fMRI measure**	**Non-candidates clinical data (36-48 M)**	**r**	**p**
R cuneus - L pericalcarine	HY	0.54	0.002

Partial correlations were assessed between fMRI measures at baseline and clinical data at study entry and at each follow-up using Pearson's correlation coefficient (r) ($p<0.05$). Analysis was adjusted for age and sex, LEDD at baseline and LEDD changes (only at each follow-up). Correlation analyses were performed controlling for multiple comparisons using Bonferroni adjustment. Abbreviations: DBS=Deep Brain Stimulation; Candidates for DBS=Patients eligible for DBS; fMRI=functional MRI; HY=Hoehn & Yahr; L=Left; M=months; Non-candidates=Patients not eligible for DBS; PD=Parkinson's disease; R=Right; UPDRS=Unified Parkinson's Disease Rating Scale.

Supplementary Table 5: Brain nodes included in the Network-based Statistical Analysis

N	Node	N	Node	N	Node
1	L thalamus	37	L precentral	73	R rostral anterior cingulate
2	L caudate	38	L precuneus	74	R rostral middle frontal
3	L putamen	39	L rostral anterior cingulate	75	R superior frontal
4	L pallidum	40	L rostral middle frontal	76	R superior parietal
5	L hippocampus	41	L superior frontal	77	R superior temporal
6	L amygdala	42	L superior parietal	78	R supramarginal
7	L accumbens	43	L superior temporal	79	R frontal pole
8	R thalamus	44	L supramarginal	80	R temporal pole
9	R caudate	45	L frontal pole	81	R transverse temporal
10	R putamen	46	L temporal pole	82	R insula
11	R pallidum	47	L transverse temporal	83	Brainstem
12	R hippocampus	48	L insula		
13	R amygdala	49	R bankssts		
14	R accumbens	50	R caudal anterior cingulate		
15	L bankssts	51	R caudal middle frontal		
16	L caudal anterior cingulate	52	R cuneus		
17	L caudal middle frontal	53	R entorhinal		
18	L cuneus	54	R fusiform		
19	L entorhinal	55	R inferior parietal		
20	L fusiform	56	R inferior temporal		
21	L inferior parietal	57	R isthmus cingulate		
22	L inferior temporal	58	R lateral occipital		
23	L isthmus cingulate	59	R lateral orbitofrontal		
24	L lateral occipital	60	R lingual		
25	L lateral orbitofrontal	61	R medial orbitofrontal		
26	L lingual	62	R middle temporal		
27	L medial orbitofrontal	63	R parahippocampal		
28	L middle temporal	64	R paracentral		
29	L parahippocampal	65	R pars opercularis		
30	L paracentral	66	R pars orbitalis		
31	L pars opercularis	67	R pars triangularis		
32	L pars orbitalis	68	R pericalcarine		
33	L pars triangularis	69	R postcentral		
34	L pericalcarine	70	R posterior cingulate		
35	L postcentral	71	R precentral		
36	L posterior cingulate	72	R precuneus		

Abbreviations: L=Left, N=Number, R=Right.

Supplementary Table 6: Head motion parameters at study entry in PD groups and healthy controls (HC) and changes over time in PD patients.

Variables	HC	Candidates for DBS	Non-candidates	p: Candidates for DBS vs HC	p: Non-candidates *vs* HC	p: Candidates for DBS *vs* Non-candidates	p different trend of change between PD groups	p similar trend of change, with or without difference between PD groups	p different but stable values over time in PD groups
**N**	60	19	41						
**Mean absolute cumulative translation [mm]**	-0.02 ± 0.19 (-0.55-0.72)	0.04 ± 0.16 (-0.18-0.42)	0.00 ± 0.28 (-0.55-1.21)	0.89	1.00	1.00	0.96	0.84	0.78
**Mean rotation [°]**	0.00 ± 0.00 (0.02-0.01)	0.00 ± 0.01 (-0.01-0.02)	0.00 ± 0.01 (-0.01-0.03)	1.00	1.00	1.00	0.50	0.80	0.60

Values are reported as mean ± standard deviation (range). Differences between PD groups and healthy controls and between PD groups at baseline were assessed using one-way ANOVA (statistical contrasts). Linear regression models were built to assess longitudinal head motion parameters changes. Head motion parameters were considered as the dependent variable into each model, which also included individual follow-up duration as covariate (independent variable). Abbreviations: Candidates for DBS=Patients eligible for DBS; HC= healthy controls; mm= millimeters; N= number; Non-candidates=Patients not eligible for DBS.

Supplementary Figure 1. Subnetworks showing altered functional connectivity in patients eligible or not to DBS relative to healthy controls (NBS analysis). The principal (or largest) connected components are shown in red; other connected components, not included in the principal connected components, are shown in green. Abbreviations: A= anterior; Candidates for DBS= patients eligible for DBS; HC= healthy controls; Non-candidates=Patients not eligible for DBS; L= left; P= posterior; R= right.

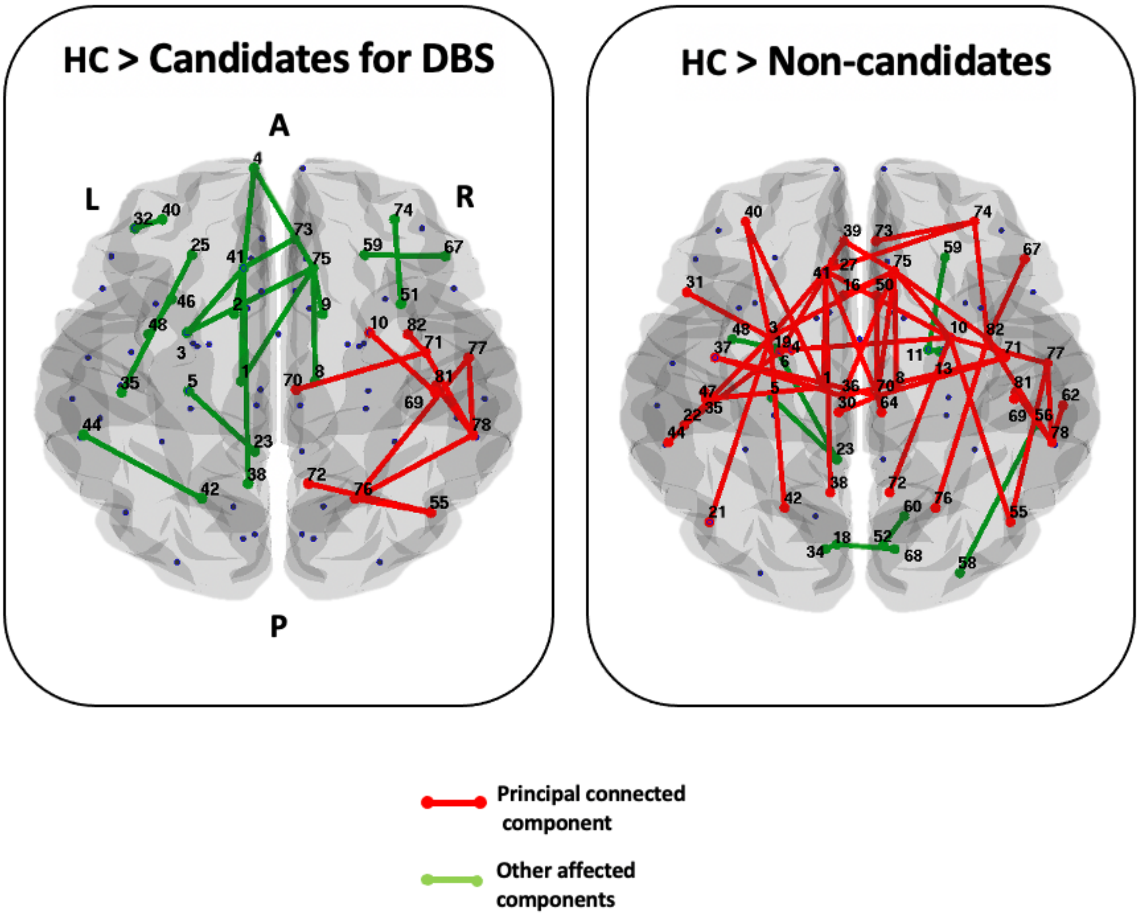

Supplementary Figure 2. Framework for the selection of Parkinson's disease patients who became candidates for DBS over time. Once defined the PD cluster of 60 patients according to inclusion and exclusion criteria (Figure 1): **a.** the UPDRS score IV, item 33 ≥2 and item 39 >2, was used to assess troublesome dyskinesia and severe motor fluctuations, respectively; subsequently, the impairment of motor activities of daily living (UPDRS II >6), the absence of severe dementia and major depression (MDS-Task Force criteria and Hamilton Depression Rating Scale) and the medications were evaluated. Patients were considered eligible for DBS in case of persistent troublesome dyskinesia and/or severe motor fluctuations causing reduced quality of daily living despite medications adjustment (no therapeutic window) over four years of follow up; and/or **b.** the UPDRS score III, item 20 and 21 ≥5, was used to assess a marked tremor; subsequently, the impairment of motor activities of daily living (UPDRS II >6), the absence of severe dementia and major depression (MDS-Task Force criteria and Hamilton Depression Rating Scale), and the medications were evaluated. Patients were considered eligible for DBS in case of refractory marked tremor impairing the quality of daily living despite medications adjustment (no therapeutic window) over four years. Abbreviations: DBS= deep brain stimulation; LEDD= levodopa equivalent daily dosage; PD= Parkinson's disease; UPDRS= Unified Parkinson's Disease Rating Scale.

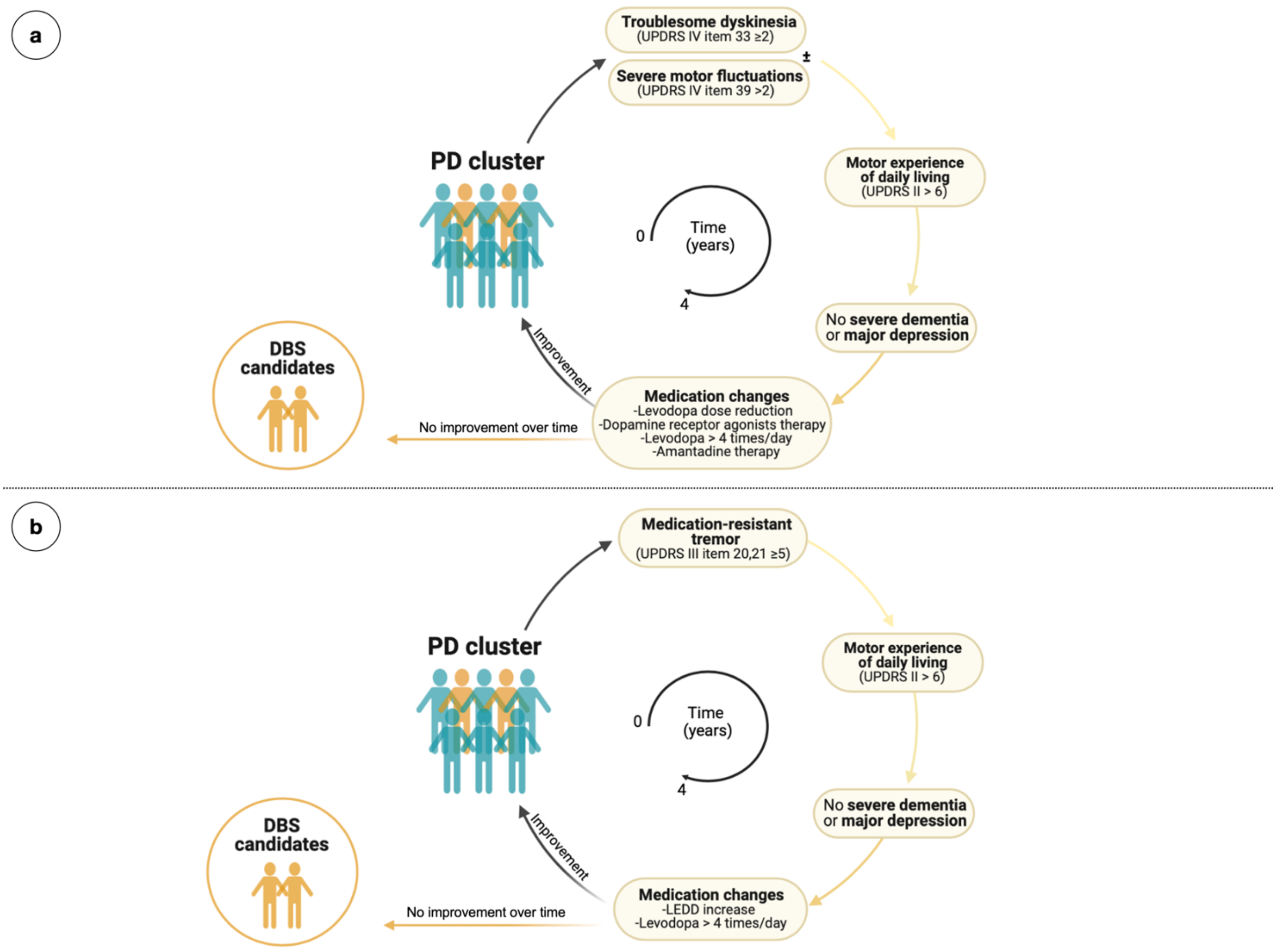
a
Troublesome dyskinesia
(UPDRS IV item 33 ≥2)
±
Severe motor fluctuations
(UPDRS IV item 39 >2)
PD cluster
Time
(years)
0
4
Motor experience
of daily living
(UPDRS II > 6)
No severe dementia
or major depression
Medication changes
-Levodopa dose reduction
-Dopamine receptor agonists therapy
-Levodopa > 4 times/day
-Amantadine therapy
Improvement
No improvement over time
DBS
candidates
b
Medication-resistant
tremor
(UPDRS III item 20,21 ≥5)
PD cluster
Time
(years)
0
4
Motor experience
of daily living
(UPDRS II > 6)
No severe dementia
or major depression
Medication changes
-LEDD increase
-Levodopa > 4 times/day
Improvement
No improvement over time
DBS
candidates
