# Supplementary material for: Functional connectivity in Parkinson’s disease candidates for deep brain stimulation
Source: NPJ Parkinsons Dis. 2022 Jan 10;8:4. doi: 10.1038/s41531-021-00268-6 (PMC8748462; doi:10.1038/s41531-021-00268-6)
Supplement: Supplementary file 2 — Reporting Summary [file 41531_2021_268_MOESM2_ESM.pdf]

nature portfolio

Corresponding author(s): Prof. Massimo Filippi

Last updated by author(s): Nov 23, 2021

Reporting Summary

Nature Portfolio wishes to improve the reproducibility of the work that we publish. This form provides structure for consistency and transparency in reporting. For further information on Nature Portfolio policies, see our Editorial Policies and the Editorial Policy Checklist.

Statistics

For all statistical analyses, confirm that the following items are present in the figure legend, table legend, main text, or Methods section.

n/a	Confirmed	
☐	☒	The exact sample size (n) for each experimental group/condition, given as a discrete number and unit of measurement
☐	☒	A statement on whether measurements were taken from distinct samples or whether the same sample was measured repeatedly
☐	☒	The statistical test(s) used AND whether they are one- or two-sided<br>*Only common tests should be described solely by name; describe more complex techniques in the Methods section.*
☐	☒	A description of all covariates tested
☐	☒	A description of any assumptions or corrections, such as tests of normality and adjustment for multiple comparisons
☐	☒	A full description of the statistical parameters including central tendency (e.g. means) or other basic estimates (e.g. regression coefficient) AND variation (e.g. standard deviation) or associated estimates of uncertainty (e.g. confidence intervals)
☐	☒	For null hypothesis testing, the test statistic (e.g. F, t, r) with confidence intervals, effect sizes, degrees of freedom and P value noted<br>*Give P values as exact values whenever suitable.*
☒	☐	For Bayesian analysis, information on the choice of priors and Markov chain Monte Carlo settings
☐	☒	For hierarchical and complex designs, identification of the appropriate level for tests and full reporting of outcomes
☐	☒	Estimates of effect sizes (e.g. Cohen's d, Pearson's r), indicating how they were calculated

Our web collection on statistics for biologists contains articles on many of the points above.

Software and code

Policy information about availability of computer code

Data collection	No software was used
Data analysis	R Statistical Software (version 4.0.3; R Foundation for Statistical Computing, Vienna, Austria)

For manuscripts utilizing custom algorithms or software that are central to the research but not yet described in published literature, software must be made available to editors and reviewers. We strongly encourage code deposition in a community repository (e.g. GitHub). See the Nature Portfolio guidelines for submitting code & software for further information.

Data

Policy information about availability of data

All manuscripts must include a data availability statement. This statement should provide the following information, where applicable:

- Accession codes, unique identifiers, or web links for publicly available datasets
- A description of any restrictions on data availability
- For clinical datasets or third party data, please ensure that the statement adheres to our policy

The dataset used and analyzed during the current study will be made available by the corresponding author upon request to qualified researchers (i.e., affiliated to a university or research institution/hospital).

Field-specific reporting

Please select the one below that is the best fit for your research. If you are not sure, read the appropriate sections before making your selection.

☒ Life sciences ☐ Behavioural & social sciences ☐ Ecological, evolutionary & environmental sciences

For a reference copy of the document with all sections, see nature.com/documents/nr-reporting-summary-flat.pdf

Life sciences study design

All studies must disclose on these points even when the disclosure is negative.

Sample size	154 patients with idiopathic Parkinson's disease were prospectively recruited. According to the reported inclusion and exclusion criteria, 60 patients were enrolled in the study (19 candidates and 41 not candidates for DBS over time). 60 age- and sex-matched healthy controls without any neurological disorders and psychiatric disorders were also recruited.
Data exclusions	Patients with incomplete MRI or clinical data were excluded.
Replication	We confirmed the replication of data.
Randomization	N/A
Blinding	N/A

Reporting for specific materials, systems and methods

We require information from authors about some types of materials, experimental systems and methods used in many studies. Here, indicate whether each material, system or method listed is relevant to your study. If you are not sure if a list item applies to your research, read the appropriate section before selecting a response.

Materials & experimental systems

n/a	Involved in the study	
☒	☐	Antibodies
☒	☐	Eukaryotic cell lines
☒	☐	Palaeontology and archaeology
☒	☐	Animals and other organisms
☐	☒	Human research participants
☒	☐	Clinical data
☒	☐	Dual use research of concern

Methods

n/a	Involved in the study	
☒	☐	ChIP-seq
☒	☐	Flow cytometry
☐	☒	MRI-based neuroimaging

Human research participants

Policy information about studies involving human research participants

Population characteristics	Epidemiological and clinical features of the enrolled subjects are detailed in the main text and tables
Recruitment	Idiopathic Parkinson's disease patients who were prospectively recruited at the Clinic of Neurology, Faculty of Medicine, University of Belgrade, Serbia
Ethics oversight	The study received approval from the ethics committee on human experimentation of Faculty of Medicine - University of Belgrade (No. 175090)

Note that full information on the approval of the study protocol must also be provided in the manuscript.

Magnetic resonance imaging

Experimental design

Design type	Resting-state fMRI
Design specifications	Total scan time was about 90-120 min for each subject. The MRI sequences are reported in the main text.
Behavioral performance measures	Neuropsychological and behavioral evaluations were performed at each visit in both Parkinson's disease patients and healthy controls. Their tests are reported in the main text.

Acquisition

Imaging type(s)	Structural and functional MRI
Field strength	1.5 T
Sequence & imaging parameters	Dual-Echo Turbo Spin-Echo (repetition time [TR]=3125 ms, echo time [TEs]=20/100 ms, echo train length [ETL]=6,44 axial slices, thickness=3.0 mm, matrix size =256×247, field of view [FOV]=240×232 mm2; voxel size=0.94×0.94×3 mm, in-plane sensitivity encoding [SENSE] parallel reduction factor, 1.5);<br>Three-dimensional (3D) sagittal T1-weighted Turbo-Field-Echo (TR=7.1 ms, TE=3.3 ms, inversion time=1000 ms, flip angle=8°, matrix size=256×256×180, FOV=256×256 mm2 , section thickness=1 mm, voxel size=1×1×1 mm);<br>Gradient-echo echo planar imaging for RS-fMRI (TR=3000 ms, TE=35 ms, flip angle=90°, matrix size=128×128, FOV=240×240 mm2, voxel size=1.88×1.88×4 mm, slice thickness=4 mm, 200 sets of 30 contiguous axial slices)
Area of acquisition	Whole brain scan was performed

Diffusion MRI ☐ Used ☒ Not used

Preprocessing

Preprocessing software	T1-weighted images were processed and parcellated using the Freesurfer suite (V 5.3 http://surfer.nmr.mgh.harvard.edu/), resulting in 83 areas, which were used to define the brain nodes for the network analysis.<br>RS-fMRI data processing was carried out using the FMRIB software library (FSLv5.0). It is reported in the main text.
Normalization	T1-weighted images were skull stripped using the Brain Extraction Tool and segmented in GM, WM, and cerebrospinal fluid (CSF) maps using the FMRIB's Automated Segmentation Tool. Resulting images were registered into the RS-fMRI native space of each subject through a 7 degree-of freedom (DOF) linear affine transformation using FMRIB's Linear Image Registration Tool.
Normalization template	N/A
Noise and artifact removal	The following FSL-standard preprocessing pipeline was applied: (1) motion correction using MCFLIRT; (2) high-pass temporal filtering (lower frequency: 0.01 Hz); (3) spatial smoothing (Gaussian Kernel of FWHM 6 mm); (4) single-session independent component analysis (ICA).
Volume censoring	The first four volumes of the fMRI data were removed to reach complete magnet signal stabilization.

Statistical modeling & inference

Model type and settings	Baseline and longitudinal MRI analysis (graph analysis and Network-Based Statistics) and correlation analysis between baseline fMRI metrics and baseline/longitudianl clinical scales.
Effect(s) tested	aseline MRI analysis: global and lobar network topological metrics were compared between groups using ANOVA models; NBS analysis compared functional connectivity between groupsusing ANOVA models.<br>Longitudinal MRI analysis: changes over time of the functional network metrics were assessed with general linear models using time as a continuous variable.<br>Correlation analysis: partial correlations were assessed between baseline fMRI metrics and baseline/longitudinal clinical scales using Pearson's correlation coefficient (p<0.05).

Specify type of analysis: ☒ Whole brain ☐ ROI-based ☐ Both

Statistic type for inference (See Eklund et al. 2016)	Graph analysis and NBS
Correction	Bonferroni correction

Models & analysis

n/a	Involved in the study
☐	☒ Functional and/or effective connectivity
☐	☒ Graph analysis
☒	☐ Multivariate modeling or predictive analysis

Functional and/or effective connectivity	NBS was used to evaluate functional connectivity changes at baseline and over time between groups.
Graph analysis	Nodal strength, Path length, Local efficiency and Clustering coefficient.
